# Correlates of physical activity and sedentary behaviour in the Thai population: a systematic review

**DOI:** 10.1186/s12889-019-6708-2

**Published:** 2019-04-16

**Authors:** Nucharapon Liangruenrom, Melinda Craike, Stuart J. H. Biddle, Kanyapat Suttikasem, Zeljko Pedisic

**Affiliations:** 1 Institute for Health and Sport, Victoria University, PO Box 14428, Melbourne, VIC 8001 Australia; 2 Institute for Population and Social Research, Mahidol University, Phutthamonthon Sai 4 Road, Salaya, Phutthamonthon, Nakhon Pathom, 73170 Thailand; 3 Australian Health Policy Collaboration, Victoria University, PO Box 14428, Melbourne, VIC 8001 Australia; 4 Institute for Resilient Regions, University of Southern Queensland, Education City, 37 Sinnathamby Boulevard, Springfield Central, QLD 4300 Australia

**Keywords:** Correlates, Physical activity, Sedentary behaviour, Systematic review, Thailand

## Abstract

**Background:**

Given the importance of knowing the potential impediments and enablers for physical activity (PA) and sedentary behaviour (SB) in a specific population, the aim of this study was to systematically review and summarise evidence on individual, social, environmental, and policy correlates of PA and SB in the Thai population.

**Methods:**

A systematic review of articles written in Thai and English was conducted. Studies that reported at least one correlate for PA and/or SB in a healthy Thai population were selected independently by two authors. Data on 21 variables were extracted. The methodological quality of the included studies was assessed using the Newcastle-Ottawa Scale.

**Results:**

A total of 25,007 records were screened and 167 studies were included. The studies reported associations with PA for a total of 261 variables, mostly for adults and older adults. For most of the variables, evidence was available from a limited number of studies. Consistent evidence was found for individual-level and social correlates of PA in children/adolescents and adults and for individual-level correlates of PA in older adults. Self-efficacy and perceived barriers were consistently associated with PA in all age groups. Other consistently identified individual-level correlates in adults and older adults included self-rated general health, mental health, perceived benefits, and attitudes towards PA. Consistent evidence was also found for social correlates of PA in adults, including social support, interpersonal influences, parent/family influences, and information support. The influence of friendship/companionship was identified as a correlate of PA only in children/adolescents.

A limited number of studies examined SB correlates, especially in older adults. The studies reported associations with SB for a total of 41 variables. Consistent evidence of association with SB was only found for obesity in adults. Some evidence suggests that male adults engage more in SB than females.

**Conclusions:**

More Thai studies are needed on (i) PA correlates, particularly among children/adolescents, and that focus on environment- and policy-related factors and (ii) SB correlates, particularly among older adults. Researchers are also encouraged to conduct longitudinal studies to provide evidence on prospective and causal relationships, and subject to feasibility, use device-based measures of PA and SB.

**Electronic supplementary material:**

The online version of this article (10.1186/s12889-019-6708-2) contains supplementary material, which is available to authorized users.

## Background

Even though physical activity (PA) has been identified as the ‘best buy’ in public health [1] and national actions for the promotion of PA have been employed in many countries [2], population levels of PA are still declining [2–4]. In contrast, time spent performing sedentary behaviour (SB) is increasing [3, 4]. SB refers to any waking activity in a sitting, reclining, or lying position with low energy expenditure [5]. In the academic literature, SB has been conceptualised in two ways: (i) as a health risk factor ‘independent’ of PA [6]; and (ii) as a part of the time-use composition consisting of sleep, SB, and PA co-dependent time-use components [7]. In both conceptualisations, SB is deemed a potentially important factor for population health. Nevertheless, there seem to be barriers to the promotion of PA and reduction of SB, especially in low- and middle-income countries. These barriers include workforce shortages in the PA/SB sector (e.g. lack of PA promoters), weak networks of collaboration with other sectors (e.g. education, sports, and transportation), the lack of effective actions, and lack of knowledge about what approaches to PA promotion and SB reduction are feasible [2, 8]. These have been major challenges in Thailand, where efforts have been made to design and implement policy-level interventions.

To develop effective programs or interventions to increase PA and reduce SB, there is a need to understand correlates of these behaviours in specific populations. Public health experts advise that this need is urgent in low- and middle-income countries [2, 9]. Moreover, given substantial differences between geographical areas in social, cultural, environmental, and economic factors, it is important to explore PA and SB correlates in specific countries so that feasible interventions can be developed and designed based on local data [10]. Studies on PA correlates in low- and middle-income countries have recently started receiving more attention [2, 9–13]. Since 1987, and especially over the last two decades, PA has been the focus of a plethora of Thai epidemiological research, and the attention has most commonly been placed on its correlates [14].

In Thailand, the data from a 2015 population-representative survey on PA and SB showed that 21-25% of Thai children and adolescents (aged 6–17 years) achieved the recommended level of PA (i.e. 60 minutes a day) [15]. In addition, more than 78% of Thai children and adolescents engaged in two or more hours of SB [15]. Around 40% of Thai adults (aged 18 and above) met the World Health Organisation (WHO) recommendations for moderate-to-vigorous PA (MVPA) [16, 17]. Interestingly, 33.8% of Thai adults reported a high level of SB and no MVPA in the past week [16]. A better understanding of what makes some Thai population groups less active than others may help tackle the problem of insufficient PA.

Like other healthy behaviours, PA and SB are influenced by many factors [9, 18–22]. However, the focus to date has mostly been on individual-level correlates, such as sex, age, attitude, and self-rated general health [9, 11, 14, 20–22]. The social-ecological approach has been widely adopted to understand the interrelationships among multiple factors that contribute to PA and SB, including individual, social, environmental, and policy factors [9, 18–22]. The full spectrum of PA/SB correlates has been analysed in several reviews, mostly in high-income countries such as the United States, Australia, and Canada [9, 20–25]. Studies from low- and middle-income countries including Thailand have rarely been included [9, 20–25].

Given the importance of knowing which variables are associated with PA and SB in a specific population, the aim of this study was to systematically review and summarise the available evidence on individual, social, environmental, and policy correlates of PA and SB in Thai children, adolescents, adults, and older adults. We also aimed to identify the key gaps in the literature on PA and SB correlates in Thailand and provide recommendations for future research.

## Methods

### Search strategy

This systematic review was conducted by following the PRISMA guidelines [26]. The primary literature search was conducted from database inception to September 2016 using the following ten bibliographic databases: Academic Search Premier; CINAHL; Health Source: Nursing/Academic Edition; MasterFILE Premier; PsycINFO; PubMed/MEDLINE; Scopus; SPORTDiscus; Web of Science; and the Networked Digital Library of Theses and Dissertations (NDLTD). The secondary literature search was conducted by using three main sources, including: the Google and Google Scholar internet search engines (using search terms in both Thai and English); references of the studies that were selected in the primary search; and the websites/databases of Thai health-related organizations and institutes including the Division of Physical Activity, Ministry of Public Health; Thai Health Promotion Foundation; Health System Research Institute; Physical Activity Research Institute; Thai National Research Repository; Thai Thesis Database; Thai NCD Network; Kasetsart University Research; Chulalongkorn University Intellectual Repository; and Institute for Population and Social Research, Mahidol University.

### Study selection and inclusion criteria

Two researchers (NL and KS) independently screened all references obtained from the search results, removed duplicates, and selected studies. The third author (ZP) resolved discrepancies about the study selections. The eligibility criteria included the following: Published peer-reviewed journal papers, theses, reports, and conference papers written in Thai or English were included, and reviews, commentaries, and editorials were excluded. Observational studies (cross-sectional, case-control, and prospective) that targeted healthy Thai people (as opposed to patients with a specific disease or health condition) of any ages were considered eligible for inclusion. To be included, the studies had to present the association of at least one variable with total PA (e.g. minutes per day or METs per week), MVPA, moderate PA (MPA), vigorous PA (VPA), meeting/not meeting PA guidelines (e.g. meeting the PA recommendation of 60 minutes of MVPA per day), domain-specific PA (e.g. recreation, transportation), and exercise participation. For SB measures, total SB or sitting time or frequency and/or duration in one or more of sedentary activities including television (TV) viewing, screen time, and computer/internet use were included. Both self-reported and devise-based measurements qualified for inclusion. Longitudinal studies that analysed PA or SB as predictors of an outcome variable, were not considered eligible for inclusion.

### Data extraction

The following data were extracted from the selected studies: (a) general bibliographical information, such as publication type and language; (b) research methods used, including sampling techniques; (c) characteristics of the study population such as sex, age, and region; (d) description of PA/SB measure, including the PA type as a dependent variable and validity information; (e) specific correlate(s) with an assigned, categorized domain such as socio-demographic, psychological, and social factors; and (f) the type of statistical analysis used in the included studies. Data were extracted separately for three age groups: children and adolescents (<18 years old); adults (between 18 and 59 years old); and older adults (>60 years old). The full extraction tables are provided in the Additional file 1.

### Data coding and pooling

To pool the results of individual studies, we used the procedure proposed by Sallis et al. [24]. The pooled associations between potential correlates and PA and SB were classified as: a) mostly positive associations (denoted by ‘+’); b) mostly negative associations (denoted by ‘-‘); or c) mostly non-significant, indeterminate, or inconsistent associations (denoted by ‘?’). The codes were determined based on the percentage of significantly positive, significantly negative, and non-significant associations, according to the rules presented in Table 1 [24]. The classification system was slightly adapted from the original categorisation used by Sallis et al. [24], to better reflect the implication of non-significant relationships. The results from the most adjusted analysis reported in a paper were used for the classification. Letters ‘M’ and ‘F’ were used to indicate findings for male and female participants when results were reported separately for sexes.

**Table 1:** Rules applied to classify correlates of physical activity

Percentage of studies (%)^a^	Code describing the association between a correlate and PA or SB^b^	Meaning of the code
0 - 59	?	Mostly non-significant, indeterminate, or inconsistent associations
60 - 100	+ or –	Mostly positive (+), or negative (–) associations

^a^Percentage of studies showing positive, negative, or non-significant association

^b^When four or more studies showed positive or negative association, the summary results were coded as ++, and --, respectively. The code “??” denoted a frequently studied correlate whose association with PA or SB was largely inconsistent across the studies

A synthesis of the findings of this review was structured by applying the social-ecological model of PA/SB, where all correlates were categorised into key components of the model including individual (e.g. socio-demographic, biological), social (e.g. interpersonal, cultural), physical environment (e.g. facilities, neighbourhood), and policy (e.g. education and workplace policies) factors. The pooled results are presented separately for children and adolescents (6-17 years), adults (18-59 years), and older adults (60 years and over). We used this threshold for the ‘older adults’ group, because, according to the Thai Labour Protection Act, the retirement age applies to adults aged 60 years and more [27].

### Risk of bias

The methodological quality of the included studies was assessed using the Newcastle-Ottawa Scale (NOS) [28]. NOS was designed to evaluate the quality of observational studies for several purposes, such as to include/exclude studies for meta-analysis, weight studies, and address areas that need methodological improvements [29]. The three aspects of studies that were assessed using the NOS tool included the selection of study groups (4 items, maximum 5 points), adjustments for potential confounders (1 item, maximum 2 points), and ascertainment of exposure and outcomes (2 items, maximum 3 points) [28, 29]. The overall score was calculated as the sum of points across the three categories. The overall scores were classified into three groups: low (<5 points), moderate (5-7 points), and high (>7 points) study quality [30].

## Results

### Characteristics of the included studies

**Fig. 1:**
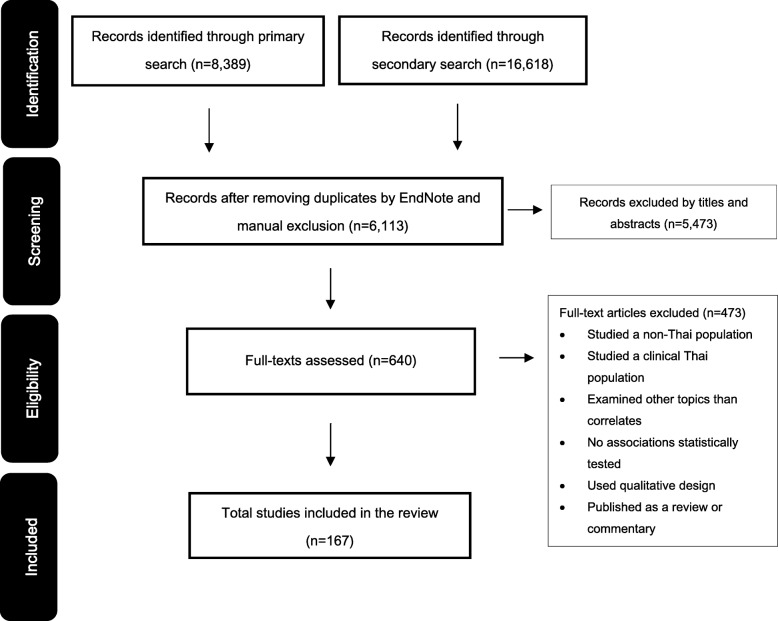
Flow diagram of study selection process

The search and study selection processes are illustrated in Fig. 1. A total of 25,007 records were identified. Of these, 167 papers met the eligibility criteria and were included in the present review. Most studies focused on PA only (76%; *n* = 127), 15 studies (9%) examined SB correlates only, and 25 studies (15%) examined correlates of both PA and SB. The included articles were published between 1993 and 2016. All studies used a cross-sectional design. The average sample size cited in the studies was 3,317 and ranged from 27 to 87,134. Two studies included only male participants (1.2%), 17 studies included only female participants (10.2%), and the remainder included both sexes (88.6%; *n* = 148). Half of the studies were conducted among adults (50.3%; *n* = 100), followed by older adults (28.6%; *n* = 57) and children/adolescents (21.1%; *n* = 42). Of these, twenty-nine studied on adults/older adults and three on adolescents/adults. Most of the included publications were articles published in peer-reviewed journals (69.5%; *n* = 116), more than a quarter were doctoral and master’s theses (26.9%; *n* = 45), and the remaining publications were reports (2.4%; *n* = 4) and conference papers (1.2%; *n* = 2).

Nearly all studies were conducted using self-report instruments to measure PA (97.6%; *n* = 163). Three studies used accelerometers (1.8%) and one study used pedometers (0.6%). Sixty-three percent of the studies used previously validated PA measures (*n* = 96). More than half of the studies measured exercise participation (54.9%; *n* = 89), followed by MVPA (19.1%; *n* = 31) and total PA (13.6%; *n* = 22). Domain-specific PA was assessed in 6.8% of the studies (*n* = 11), which mainly included recreation and household PA. The remaining studies assessed VPA (3.1%; *n* = 5) and MPA (2.5%; *n* = 4). Presenting correlates of each PA type separately are available in the Additional file 2: Tables S1-S22.

SB was assessed using self-report instruments in all but one study. Most instruments used to measure SB had been previously validated (62.5%; *n* = 25). Watching television was the main SB independently investigated in several studies (30.6%; *n* = 15), followed by total SB time (20.4%; *n* = 10). Other individual sedentary activities included computer and internet use that were examined in 16.3% (*n* = 8) and 10.2% (*n* = 5) of the studies respectively. Screen time, which refers to TV viewing and computer use combined, was assessed in 12.2% of the studies (*n* = 6). The remainder observed the total duration of sitting during leisure time (6.1%; *n* = 3) and at work (4.1%; *n* = 2).

### Methodological quality

The median overall score of the included studies on the NOS was 6 (‘moderate quality’). Twenty-three studies were categorised as ‘high quality’, 34 were considered ‘low quality’, while the remainder were of moderate quality (*n* = 110). Among the high-quality studies, seven studies dealt with children and adolescents, seven focused only on adults, two only on older adults, and seven on both adults and older adults. It was, therefore, not considered appropriate to perform a sensitivity analysis using only findings from high-quality studies, due to the fact that too few of these studies were available. The results of the quality assessment for all the included studies can be found in the Additional file 3.

### Physical activity correlates

**Fig. 2:**
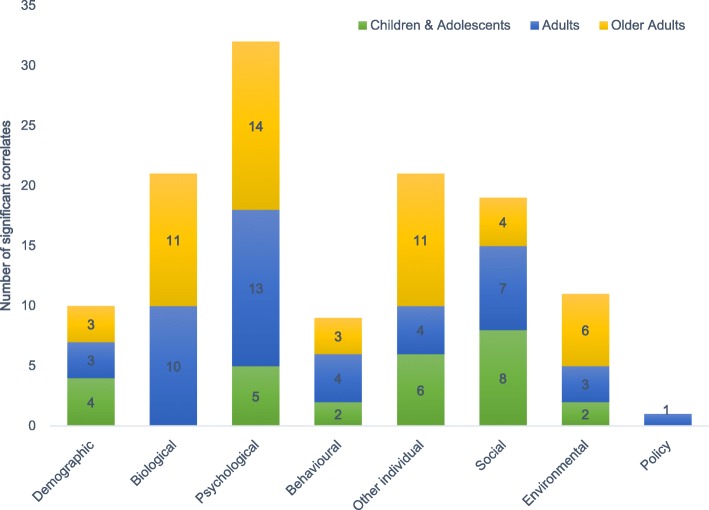
The number of significant correlates of physical activity across different categories

The included studies reported associations with PA for a total of 261 variables [31–181]. Almost half of the variables were significantly associated with PA (47.5%; *n* = 124). Multiple factors were assessed, including individual, social, physical, and policy environment variables. The most frequently studied factors were those at the individual level (81.6%; *n* = 213). The statistically significant correlates most often included psychological factors, followed by biological, demographic, and health behavioural and lifestyle factors (Fig. 2).

#### Correlates of children’s and adolescents’ physical activity

A total of 52 potential PA correlates were studied in Thai children and adolescents (Table 2). Consistent evidence of association with higher PA was found for the following individual-level factors: younger age, being a male, higher self-efficacy, and lower perceived barriers. Consistent evidence of association with higher PA was found for the social factor of greater friendship/companionship influences. No consistent evidence was found for environmental and policy correlates of PA.

**Table 2:** Summary of evidence on physical activity (PA) correlates in Thai children and adolescents (6 – 17 years old)

Correlates	Relationship with PA			Summary Code
	Negative/Inverse (-)	Positive (+)	Non-significant (?)	
*Individual Level*				
*Demographic factors*				
– Age	97, 135 (M, F), 183		158 (M, F)	--
– Sex (+ denotes males are more active, - denotes females are more active)		51, 55, 97, 111, 118, 122, 126, 135, 158, 167, 178, 183	169, 189	++
– Household income	96, 178			-
– School type (+ denotes more PA in mixed-gender schools, - denotes more PA in single-gender schools)		55		+
– School grade	80		51, 118, 167, 189	??
– Parents’ occupation			189	?
– Municipality (+ denotes more PA in an urban place of residence, - denotes more PA in a rural place of residence)		169	135 (M, F), 64	?
– Household location within Bangkok			178	?
*Biological factors*				
– Body weight			135 (M, F)	?
– Body mass index (BMI)	97, 178		33, 51, 135 (M, F), 158	??
– Parents’ BMI			178	?
– Underweight			110	?
– Overweight	110, 157	111 (F)	52, 111 (M), 125 (F)	?
– Obesity	110, 130 (M), 157		125 (F), 130 (F), 169	?
– Body fat (%)		135 (M)	135 (F)	?
– Height	135 (M, F)		77 (M, F)	?
– Low-density lipoprotein cholesterol (LDL-C) level			158	?
– Systolic blood pressure			158	?
– Diastolic blood pressure			158	?
– Dietary fat intake			158	?
*Physical health*				
– Asthma	157			-
– Hypertension	157			-
*General health*				
– Self-rated general health		121 (M, F)		+
– Child’s health status as perceived by parents			45	?
*Psychological factors*				
– Self-efficacy		45, 51, 80, 123	167	++
– Perceived benefits of PA/exercise		51, 80	38 (M, F), 167	?
– Perceived barriers for PA/exercise	38 (F), 51, 80, 123		38 (M), 167	--
– Attitudes towards PA/exercise		178	167	?
– Self-esteem			167	?
– Cues to action			38 (M, F)	?
– Being bullied	129 (M)		129 (F)	?
– Expected outcomes of PA/exercise			167	?
– Body dissatisfaction	55			-
– Enjoyment of PA/exercise		80, 183	178	+
– Resilience		118		+
*Physical skills, abilities, and fitness*				
– Perceived physical competence		183		+
*Health behavioural and lifestyle factors*				
– Past PA/exercise experience		51, 80	167	+
– TV viewing	178			-
*Knowledge*				
– Knowledge about PA/exercise		167		+
– Parents’ knowledge about school-aged children’s PA			45	?
*Academic/school performance*				
– Grade point average	157			-
*Social environment*				
*Social and cultural factors*				
– Social support		123, 178	167	+
– Parent/family influences		80, 183	178	+
– Friendship/companionship influences		80, 122 (M, F), 183		++
– Involvement with friends		122 (M, F)		+
– Ease in making friends		122 (M, F)		+
– Teacher influences		80		+
– General interpersonal influences		51		+
– Information support (e.g. social media)		167		+
*Physical environment*				
*Environmental factors*				
– Environmental support (e.g. situational influences)		51, 80, 178		+
– Supportive physical environment (e.g. facilities, supplies)		178	167	?
– Supportive neighbourhood environment		178		+

(+) Mostly positive associations, (-) Mostly negative associations, (?) Mostly non-significant, indeterminate, or inconsistent associations, (M) Male, (F) Female

Some evidence supported associations between higher PA and the following individual-level factors: lower household income, going to mixed-gender schools (compared with single-gender schools), higher self-rated general health, greater enjoyment of PA/exercise, more past PA/exercise experience, higher resilience, higher perceived physical competence, greater knowledge of PA/exercise, not having asthma and hypertension, lower body dissatisfaction, lower duration of TV viewing, and lower grade point average. Some evidence supported associations between higher PA and the following social factors: greater parent/family influences, more involvement with friends, ease in making friends, better social supports, greater teacher influences, greater general interpersonal influences, and better information support. Some evidence supported associations between higher PA and the following environmental factors: better environmental supports, and better neighbourhood environment. No evidence was found for policy correlates of PA.

The associations between PA and school grade and body mass index (BMI) were mostly non-significant or largely inconsistent.

#### Correlates of adults’ physical activity

In total, 120 potential correlates of PA were studied in Thai adults (Table 3). Consistent evidence of association with higher PA was found for the following individual-level factors: higher self-rated general health, better mental health, positive attitudes towards PA/exercise, higher self-efficacy, higher perceived benefits of PA/exercise, lower perceived barriers for PA/exercise, and more spare time. Consistent evidence of association with higher PA was found for the following social factors: better social support, greater general interpersonal influences, greater parent/family influences, and better information support. No consistent evidence was found for environmental and policy correlates of PA.

**Table 3:** Summary of evidence on physical activity (PA) correlates in Thai adults (18 – 59 years old)

Correlates	Relationship with PA			Summary Code
	Negative/Inverse (-)	Positive (+)	Non-significant (?)	
*Individual Level*				
*Demographic factor*				
– Age		37, 58, 59, 107, 124, 140, 172	58, 72, 81, 112, 116, 117, 155, 176, 190, 197	??
– Sex (+ denotes males are more active, - denotes females are more active)	50, 100, 155, 196	31, 83, 87, 100, 107, 113, 116, 117, 131, 132, 136, 150, 159, 165, 180, 181, 184, 186, 190, 193, 195	36, 44, 58, 59, 72, 92, 95, 112, 124, 140, 172, 176, 194, 197	??
– Municipality (+ denotes more PA in an urban place of residence, - denotes more PA in a rural place of residence)	58		58	?
– Marital status (+ denotes singles are more active)		155	58, 59, 72, 81, 101, 112, 117, 172, 190, 197	??
– Education level	58, 116, 117, 181	59, 155, 190	58, 72, 101, 112, 172, 197	??
– University year	165	137, 186	36, 95, 179, 193, 195	??
– Faculty*		44, 87, 165, 194, 195, 196	92, 95, 159, 179, 186	??
– Household income	117	58, 92	58, 59, 72, 101, 172, 179, 181, 190, 195, 197	??
– Occupation (+ denotes unemployed are more active)		155, 190	58, 59, 101, 117	??
– Region (+ denotes Central including Bangkok residents are more active, - denotes residents of other provinces are more active)	134	155	172	?
– Early-life (0-5) urban exposure			35	?
– Number of family members			101	?
– Student residency (+ denotes residents of university dorms are more active, - denotes students living in other accommodation types are more active)		195	95, 181	?
– Hometown (urban/rural)			92	?
– Campus/working location*		163, 197	44, 112, 159, 194	??
– Having a dependant			81	?
– Years of working experience		172		+
– Working position			112, 172, 197	?
– Working type			197	?
– Duration of health club membership			117	?
– Having a ‘dream job’		92		+
– Extra job (+ denotes yes, - denotes no)			112	?
– Ever attended a workshop on exercise (+ denotes yes, - denotes no)		124		+
*Biological factors*				
– Body mass index (BMI)	179	180	58, 109, 181, 197	??
– Underweight		131 (F), 150	131 (M)	+
– Overweight	150		65 (M, F), 114, 131 (M, F), 146 (M, F)	??
– Obesity	46 (M, F), 47 (M, F), 86, 114, 150		65 (M, F), 128, 131 (M, F)	??
– Waist circumference			197	?
– Body fat (%)			109	?
– Bone mineral density (BMD)		134, 138, 160 (M, F)	74, 90, 145	?
– Calcaneal stiffness index		160 (M, F)	160 (M, F)	?
– Skeletal muscle mass percent			143	?
– Dietary calcium intake		138		+
– Adequate serum vitamin D levels			143	?
– Sunlight exposure		138		+
– Total cholesterol (TCH) level			60 (M, F), 107	?
– Triglycerides (TG) level	60 (M, F), 107			-
– HDL-cholesterol level		60 (M, F), 107		+
– LDL-cholesterol level			107	?
– Total cholesterol: HDL-C ratio	60 (M, F)			-
– High TCH			60 (M, F)	?
– High TG	60 (M, F)			-
– Low HDL-C			60 (M, F)	?
– High TCH: HDL-C ratio			60 (M, F)	?
– Resting heart rate	107, 166 (M, F)			-
– Mean heart rate			166 (M, F)	?
– Predicted maximum heart rate (%)			166 (M, F)	?
– Systolic blood pressure			107, 166 (M, F)	?
– Diastolic blood pressure			107, 166 (M, F)	?
– Fasting plasma glucose			107	?
– VO_2_max		166 (M, F)		+
– FEV1 (Abnormal lung function)			166 (M, F)	?
– FVC (Pulmonary function test)			166 (M, F)	?
– FEV1/FVC (%)			166 (M, F)	?
– Hematocrit level	73			-
– Hypercholesterolemia			103 (M, F)	?
*Physical health*				
– History of sickness/Underlying illness/Co-morbid diseases (+ denotes yes, - denotes no)			59, 101, 112, 181, 195, 197	??
– Hypertension			69	?
– Metabolic syndrome			136 (M, F)	?
– Osteoporosis		138		+
– Musculoskeletal symptoms	63, 144		85, 89, 133	?
– Relative appendicular skeletal muscle mass (RASM)		105 (F)	105 (M)	?
– Dysmenorrhea			57 (F)	?
– Age-related macular degeneration (AMD)	88			-
*General health*				
– Self-rated general health		87, 94, 175, 194, 197	159	++
*Psychological factors*				
– Mental health		32, 94, 175, 190		++
– Attitudes towards PA/exercise		49, 50 (M, F), 117, 154, 165, 186, 188, 190, 197	44, 70, 82, 112	++
– Overall health belief		170		+
– Self-efficacy		81, 83, 87, 140, 159, 172, 187, 194	72, 101, 137	++
– Perceived benefits of PA/exercise		50 (M, F), 83, 87, 101, 137, 172, 175, 194	59, 72, 159, 170, 187	++
– Perceived barriers for PA/exercise	72, 83, 87, 159, 170, 172, 176, 194		62, 101, 187	--
– Outcome expectancies		81, 140		+
– Intention to PA/exercise		140, 179		+
– Enjoyment of PA/exercise		59		+
– Perceived exercise-related effect	140		72, 170	?
– Career satisfaction		182		+
– Motivation for PA/exercise		83	76, 195	?
– External regulation			76	?
– Extrinsic motivation	76			-
– Intrinsic motivation		76		+
– Commitment to PA/exercise			151	?
– Stress level	179			-
– Sense of coherence			108	?
– Identity achievement			108	?
– Score on memory and intelligence tests			32	?
*Physical skills, abilities, and fitness*				
– Physical and functional fitness (e.g. walking distance, leg strength)		94, 166 (M, F)		+
*Health behavioural and lifestyle factors*				
– Smoking			78 (M, F), 153	?
– Alcohol consumption		177 (M)	177 (F)	?
– Healthy dietary habits		119		+
– Past PA/exercise experience		72		+
– Being a university athlete			137, 165	?
– Availability and/or engagement in enjoyable sports		115, 137		+
– Loving watching sports			115	?
– Having a favourite athlete			115	?
– Sedentary time	58		127	?
– Spare time		49, 59, 82, 115		++
– Monosodium glutamate (MSG) intake			71	?
*Knowledge*				
– Knowledge about PA/exercise	154	44, 50 (M, F), 59, 101, 117, 188, 194	49, 82, 87, 112, 159, 165, 195, 197	??
*Academic/school performance*				
– Grade point average			179	?
*Social environment*				
*Social and cultural factors*				
– Social support		50 (M, F), 87, 154, 159, 165, 180, 194, 197	101	++
– Cultural support		180		+
– General interpersonal influences	151	49, 82, 154, 187, 197	117	++
– Parent/family influences		81, 83, 137, 172	72, 117	++
– Family members’ involvement in PA/exercise		137		+
– Friendship/companionship influences		81		+
– Friends’ involvement in PA/exercise			115	?
– Information support (e.g. from media)		49, 154, 159, 194, 197	82	++
– Religion (+ denotes Buddhists are more active, - denotes Muslims are more active)		184	95	?
*Physical environment*				
*Environmental factors*				
– Environmental support (e.g. situational influences)		137	72, 151, 195	?
– Supportive physical environment (e.g. facilities, supplies)		81, 82, 87, 154	49, 101, 197	?
– Supportive neighbourhood environment			81	?
– Urban environment	35, 100 (M, F)			-
– Distance to work			58	?
– Distance to the nearest shopping place	58			-
– Distance to the nearest recreation facility			58	?
– Distance to the nearest religious establishment			58	?
– Convenience of travel			115	?
– Month of the year (+ denotes higher PA in later months of a calendar year)		109		+
*Policy*				
*Policy attribute*				
– Supportive education policies		82, 154, 165	49	+
– Supportive workplace policies			197	?

(+) Mostly positive associations, (-) Mostly negative associations, (?) Mostly non-significant, indeterminate, or inconsistent associations, (M) Male, (F) Female, Faculty* and Campus/working location* variables - due to a number of categories of these variables, we used only + (to denote any significant association of specific faculties or campuses/working locations) and ? (to denote a non-significant association) codes.

Some evidence supported associations between higher PA and the following individual-level factors: being underweight, higher HDL-cholesterol level, higher VO_2_Max, lower triglycerides (TG) level, lower total cholesterol: HDL-C ratio, lower risk of having high TG, lower resting heart rate, lower haematocrit level, lower age-related macular degeneration, more years of working experience, having a ‘dream job’, ever attended a workshop on exercise, higher dietary calcium intake, higher sunlight exposure, having osteoporosis, greater overall health belief, higher career satisfaction, higher intrinsic motivation, healthier dietary habits, past PA/exercise experience, more enjoyment of PA/exercise, higher outcome expectancies, greater intention to take part in PA/exercise, better physical and functional fitness, engaging in enjoyable sports, lower extrinsic motivation, and lower stress level. Some evidence supported associations between higher PA and the following social factors: better cultural support, family members’ involvement in PA/exercise, and greater friendship/companionship influences. Some evidence of association with higher PA was found for the following environmental factors: less exposure to urban environment, later months of a calendar year (compared with the other months), and shorter distance to shopping place. Some evidence supported association between higher PA and the policy factor of better supportive education policies.

The associations between PA and age, sex, marital status, education level, university year, faculty, household income, occupation, campus/working location, BMI, being overweight, obesity, history of sickness, and knowledge of PA/exercise were mostly non-significant or largely inconsistent.

#### Correlates of older adults’ physical activity

A total of 89 potential correlates of PA were studied in Thai older adults (Table 4). Consistent evidence of association with higher PA was found for the following individual-level factors: higher self-rated general health, better mental health, positive attitudes towards PA/exercise, higher self-efficacy, higher perceived benefits of PA/exercise, lower perceived barriers for PA/exercise, higher outcome expectancies, greater knowledge of PA/exercise, and better physical and functional fitness. No consistent evidence was found for social, environmental, and policy correlates of PA.

**Table 4:** Summary of evidence on physical activity (PA) correlates in Thai older adults (60 years old and above)

Correlates	Relationship with PA			Summary Code
	Negative/Inverse (-)	Positive (+)	Non-significant (?)	
*Individual Level*				
*Demographic factors*				
– Age	104, 139, 164, 191, 192	37, 58, 59, 124, 140	58, 66, 116, 117, 142, 155, 162, 174, 177, 185	??
– Sex (+ denotes males are more active, - denotes females are more active)	66, 100, 155	31, 42, 100, 116, 117, 142, 152, 162, 174, 184, 185	39, 58, 59, 68, 124, 139, 140, 164, 176, 191	??
– Municipality (+ denotes more PA in an urban place of residence, - denotes more PA in a rural place of residence)	58	192	58	?
– Marital status (+ denotes singles are more active)		66, 155	58, 59, 117, 139, 164, 174, 185, 191	??
– Education level	58, 116, 117	40, 59, 139, 142, 155, 162, 164, 185, 192	39, 58, 66, 174, 191	??
– Household income	66, 117	39, 40, 58, 139, 185, 192	58, 59, 142, 162	??
– Occupation (+ denotes unemployed are more active)		155	58, 59, 117	?
– Region (+ denotes Central including Bangkok residents are more active, - denotes residents of other provinces are more active)	134	155		?
– Daily duties (+ denotes individuals performing daily duties regularly are more active, - denotes individuals performing daily duties occasionally are more active)	162			-
– Senior-citizen club membership (+ denotes yes, - denotes no)		139, 191	66	+
– Duration of health club membership			117	?
– Ever attended a workshop on exercise (+ denotes yes, - denotes no)		124		+
*Biological factors*				
– Body mass index (BMI)			58, 66	?
– Overweight			147 (M, F)	?
– Obesity	47 (M, F)			-
– Bone mineral density (BMD)	42 (F)	99, 134, 138	42 (M)	+
– Skeletal muscle mass (%)			143	?
– Dietary calcium intake		138		+
– Adequate serum vitamin D levels			143	?
– Sunlight exposure		138		+
– Total cholesterol (TCH) level			60 (M, F)	?
– Triglycerides (TG) level	60 (M, F)			-
– HDL-cholesterol level		60 (M, F)		+
– Total cholesterol: HDL-C ratio	60 (M, F)			-
– High TCH			60 (M, F)	?
– High TG	60 (M, F)			-
– Low HDL-C			60 (M, F)	?
– High TCH: HDL-C ratio			60 (M, F)	?
– Resting heart rate	166 (M, F)			-
– Mean heart rate			166 (M, F)	?
– Predicted maximum heart rate (%)			166 (M, F)	?
– Systolic blood pressure	161		166 (M, F)	?
– Diastolic blood pressure	161		166 (M, F)	?
– VO_2_max		166 (M, F)		+
– FEV1 (Abnormal lung function)			166 (M, F)	?
– FVC (Pulmonary function test)			166 (M, F)	?
– FEV1/FVC (%)			166 (M, F)	?
– Hematocrit level	73			-
– Hypercholesterolemia			103 (M, F)	?
*Physical health*				
– History of sickness/Underlying illness/Co-morbid diseases (+ denotes yes, - denotes no)	66		59, 162	?
– Having health symptoms (i.e. fatigue, weight loss, sleep disorders) (+ denotes yes, - denotes no)	162			-
– Osteoporosis	138			-
– Age-related macular degeneration (AMD)	88			-
*General health*				
– Self-rated general health		98, 139, 164, 169, 175	192, 185	++
– Health-related quality of life		41, 48, 56		+
– General oral-health status (e.g. number of teeth, and oral malodour)			152	?
– Periodontal disease	152			-
– Saliva flow rate		152		+
*Psychological factors*				
– Mental health		32, 43, 98, 175	43	++
– Attitudes towards PA/exercise		79, 117, 188, 191		++
– Self-efficacy		79, 98, 104, 139, 140, 162, 185	39, 53, 174	++
– Perceived benefits of PA/exercise		40, 79, 139, 174, 177, 192	39, 59	++
– Perceived barriers for PA/exercise	39, 40, 79, 139, 176		53, 174	--
– Outcome expectancies		79, 104, 140, 162		++
– Intention to PA/exercise		79, 140		+
– Perceived control		79		+
– Subjective norms		79		+
– Enjoyment of PA/exercise			59	+
– Perceived health risks of exercise	140			-
– Life satisfaction		120		+
– Career satisfaction		182		+
– Motivation for PA/exercise			174	?
– Score on memory and intelligence tests			32	?
– Commitment to an exercise plan		53		+
*Physical skills, abilities, and fitness*				
– Ability to do daily activities		192		+
– Physical and functional fitness (e.g. walking distance, leg strength)		43, 34, 93, 166 (M, F)		++
– Score on Timed Up and Go test	93			-
*Health behavioural and lifestyle factors*				
– Smoking	66		78 (M, F), 153	?
– Alcohol consumption	66	177 (M)	177 (F)	?
– Sedentary time	58			-
– Spare time		59		+
– Oral-health behaviours (i.e. tooth brushing, and regular dental visits)		152		+
*Knowledge*				
– Knowledge about PA/exercise		59, 117, 188, 191	162	++
*Social environment*				
*Social and cultural factors*				
– Social support		53, 192	98, 162, 174	?
– General interpersonal influences		139	117	?
– Parent/family influences		191, 192	39, 104	?
– Friendship/companionship influences		191, 192	104	+
– Hospital staff support		191		+
– Information support (e.g. from media)		164		+
– Religion (+ denotes Buddhists are more active, - denotes Muslims are more active)		184, 192		+
*Physical environment*				
*Environmental factors*				
– Supportive physical environment (e.g. facilities, supplies)		39, 192	117	+
– Supportive neighbourhood environment		39, 40, 104	98	+
– A sense of community		98		+
– Urban environment	100 (M, F)			-
– Residential community (+ denotes rural community is more active, - denotes residential home is more active)		68		+
– Distance to work			58	?
– Distance to the nearest shopping place	58			-
– Distance to the nearest recreation facility			58	?
– Distance to the nearest religious establishment			58	?

(+) Mostly positive associations, (-) Mostly negative associations, (?) Mostly non-significant, indeterminate, or inconsistent associations, (M) Male, (F) Female

Some evidence of association with higher PA was found for the following individual-level factors: being a senior-citizen club member, being a Buddhist (when compared with being a Muslim), not being obese, lower triglyceride (TG), lower total cholesterol: HDL-C ratio, less likely to have high TG, lower resting heart rate, higher bone mineral density (BMD), higher HDL-cholesterol, higher VO_2_max, better health-related quality of life, ever attended a workshop on exercise, higher dietary calcium intake, higher sunlight exposure, better saliva flow rate, less daily duty, lower hematocrit level, less likely to have abnormal symptoms, not having osteoporosis, not having age-related macular degeneration, not having periodontal disease, not perceiving exercise-related effect, lower score on Timed Up and Go test, lower sedentary time, greater perceived control, higher subjective norms, higher life satisfaction, higher career satisfaction, commitment to a plan of exercise, ability to do daily activities, greater enjoyment of PA/exercise, more spare time, better oral-health behaviours, and greater intention to PA/exercise. Some evidence supported associations between higher PA and the following social factors: greater friendship/companionship influences, better hospital staff support, and better information support. Some evidence supported associations between higher PA and the following environmental factors: better physical environment, better supportive neighbourhood environment, a sense of community, less exposure to urban environment, living in a residential community, and shorter distance to shopping place. No evidence was found for policy correlates of PA.

The associations between PA and age, sex, marital status, education level, and household income were mostly non-significant or largely inconsistent.

### Sedentary behaviour correlates

The included studies reported associations with SB for a total of 41 variables [32, 42, 45, 46, 56, 67, 79, 80, 83, 89, 93, 97, 102, 109, 116, 117, 119, 120, 122, 124, 126, 143, 155, 161, 162, 182–196]. More than half of them were significantly associated with SB (53.7%; *n* = 22). In older adults, the included studies investigated only potential SB correlates at the individual level, while they also examined social factors in children and adolescents, and in adults, also environmental factors.

#### Correlates of children’ and adolescents’ sedentary behaviour

A total of 19 potential correlates of SB were studied in Thai children and adolescents (Table 5). Some evidence of association with higher SB was found for older age/higher school grade, higher body weight, higher BMI, more physical pain, less participation in sports, more time spent with family, and more participation in extracurricular activities. Non-significant associations with SB were consistently found for sex, being overweight, and obesity.

**Table 5:** Summary of evidence on sedentary behaviour (SB) correlates in Thai children and adolescents (6 – 17 years old)

Correlates	Relationship with SB			Summary Code
	Negative/Inverse (-)	Positive (+)	Non-significant (?)	
*Individual Level*				
*Demographic factors*				
– Age/School grade		118, 147	91	+
– Sex (+ denotes higher SB in males , - denotes higher SB in females )	147	67, 75, 91, 111, 118, 141	67, 91, 111, 118, 126, 135	??
– Household income		96	75	?
– Parents’ marital status			75	?
– Parents’ education level			91	?
– Municipality (+ denotes higher SB in an urban place of residence, - denotes higher SB in a rural place of residence)	135 (M)	106	67, 106, 135 (F)	?
*Biological factors*				
– Body weight		61		+
– Body mass index (BMI)		147		+
– Overweight		111 (F), 171, 157	111 (M, F), 125 (F), 157	??
– Obesity		47 (M, F), 130 (M), 148, 156, 157, 169	33, 96 (F), 130 (F), 157, 173	??
*Physical health*				
– Asthma			157	?
– Hypertension		157	157	?
– Physical pain		61		+
*Health behavioural and lifestyle factors*				
– Physical activity participation			178	?
– Playing sports	61			-
*Academic/school performance*				
– Grade point average	54, 75, 91	61	91, 157	?
*Social environment*				
*Social and cultural factors*				
– Time spent with family		61		+
– Good relationship with friends			61	?
– Participation in extracurricular activities		61		+

(+) Mostly positive associations, (-) Mostly negative associations, (?) Mostly non-significant, indeterminate, or inconsistent associations, (M) Male, (F) Female

#### Correlates of adults’ sedentary behaviour

In total, 17 potential correlates of SB were studied in Thai adults (Table 6). A consistent association with SB was found for obesity. Some evidence of association with higher SB was found for being a male, higher education level, low back pain, higher alcohol consumption, lower grade point average, more musculoskeletal symptoms, heavy Internet use, less transport PA, less recreation PA, and more exposure to an urban environment.

**Table 6:** Summary of evidence on sedentary behaviour (SB) correlates in Thai adults (18 – 59 years old)

Correlates	Relationship with SB			Summary Code
	Negative/Inverse (-)	Positive (+)	Non-significant (?)	
*Individual Level*				
*Demographic factors*				
– Age		102	84	?
– Sex (+ denotes higher SB in males, - denotes higher SB in females)	84	75, 102, 141		+
– Household income		149	75	?
– Parents’ marital status			75	?
– Education level		102		+
*Biological factors*				
– Underweight			131 (M, F)	?
– Overweight			131 (M, F)	?
– Obesity		46 (M, F), 47 (M, F), 86	46 (M), 131 (M, F)	++
*Physical health*				
– Musculoskeletal symptoms		85		+
– Low back pain		89, 133 (F)	89	+
– Relative skeletal muscle mass of limbs	105 (F)		105 (M)	?
*Health behavioural and lifestyle*				
– Having transport and recreation physical activity	58			-
– Heavy internet use		127		+
– Monosodium glutamate (MSG) intake			71	?
– Alcohol consumption		177 (M, F)		+
*Academic/school performance*				
– Grade point average	75, 84, 149			-
*Physical environment*				
*Environmental factors*				
– Urban environment		100		+

(+) Mostly positive associations, (-) Mostly negative associations, (?) Mostly non-significant, indeterminate, or inconsistent associations, (M) Male, (F) Female

#### Correlates of older adults’ sedentary behaviour

Only five potential correlates of SB were studied in Thai older adults (Table 7). No consistent evidence from multiple studies was found on SB correlates in this age group. Some evidence of an association with higher SB was found for obesity, higher alcohol consumption, worse mental health, less active transport, and less recreational PA.

**Table 7:** Summary of evidence on sedentary behaviour (SB) correlates in Thai older adults (60 years old and above)

Correlates	Relationship with SB			Summary Code
	Negative/Inverse (-)	Positive (+)	Non-significant (?)	
*Individual Level*				
*Psychological factors*				
− Mental health	43			-
*Biological factors*				
− Obesity		47 (M, F)		+
*Physical skills, abilities, and fitness*				
− Functional fitness	43		43	?
*Health behavioural and lifestyle factors*				
− Engaging in transport and recreation physical activity	58			-
− Alcohol consumption		177 (M, F)		+

(+) Mostly positive associations, (-) Mostly negative associations, (?) Mostly non-significant, indeterminate, or inconsistent associations, (M) Male, (F) Female

## Discussion

### Physical activity correlates

A range of factors potentially associated with PA levels in the Thai population were identified at the individual, social, environmental, and policy levels. However, consistent evidence of association with PA was found only for individual-level and social correlates in children/adolescents and adults and for individual-level correlates in older adults. The summary findings suggest that PA promotion strategies in Thailand need to address several intrapersonal and interpersonal factors and may need to be tailored specifically for different age groups. The lack of consistent evidence from multiple studies for environmental and policy correlates may partially be explained by the fact that most of the included studies assessed individual-level and social correlates only. Future research should, therefore, place more focus on examining potential environmental and policy correlates of PA in the Thai population. Our findings also suggest that correlates of PA in Thailand may be different than in some other countries, calling for more focused, individual-country reviews of this kind.

In the current review, we found consistent evidence for the associations of younger age and male sex with higher PA only in children and adolescents. Among Thai adults and older adults these associations were inconsistent. A previous review for low- and middle-income countries [2] reported “mixed or weak” associations of gender and age with PA, whilst reviews for high-income countries [9, 23, 25] reported consistent associations. This might suggest that socioeconomic context in a given country may play a role in shaping the relationships of age and gender with PA. This should be further explored in future reviews of PA correlates in low- and middle-income countries and individual studies on gender- and age-specific determinants of PA. Given a relatively large number of studies included in the current review examining the association of PA with age and sex, the lack of consistent evidence in adults and older adults may suggest that the situation in Thailand is indeed different than in some other countries. For most other demographic variables, evidence on their association with PA was scarce or inconsistent in all age groups. More research is needed to understand which demographic characteristics are associated with higher PA levels in Thailand.

Evidence has strongly supported associations between different aspects of health and PA regardless of age [1, 197–200]. Whilst we found evidence on the association of PA with self-reported general health in all age groups and with mental health in adults and older adults, evidence for most other, specific health variables and biological factors was scarce or inconsistent. Moreover, it should be noted that some of the identified health-related variables associated with PA may be outcomes of PA rather than factors affecting PA [201]. For example, it may be that in Thai adults high PA improves general health status, whilst it may also be that healthier Thai adults are more likely to engage in PA. The causality might as well be bidirectional [201]. From the cross-sectional studies included in this review, it was not possible to conclude about the causal direction of the relationships. Clearly, more research is needed on biological and health-related correlates of PA in the Thai population. Furthermore, a large proportion of non-significant associations found between obesity and PA in Thai adults is in accordance with findings of most previous, non-country-specific reviews [6]. Although it may seem reasonable to assume that obese people are less likely to engage in PA, and that more physically active people are less likely to get obese, this association is clearly not so straightforward. Multiple other factors, particularly diet, need to be considered to understand the potential association between PA and obesity.

Previous reviews have identified physical skills, abilities, and fitness as important correlates of PA in all age groups [25, 202, 203]. Based on our review, it seems that this is also the case in the Thai context. It should be noted, however, that findings for nearly every variable in this category are based on results from only one study. Furthermore, findings for behavioural and lifestyle correlates of PA were mixed. We found evidence suggesting that higher PA is associated with past PA/exercise experience in children/adolescents and adults and knowledge of PA/exercise in children and older adults. Providing exercise instructions and opportunities to gain experience in PA/exercise might, therefore, be an effective way to increase PA participation in the Thai population. We also found that having more spare time may be associated with higher PA in adults and older adults. Time management interventions aimed at achieving balanced time use, to ensure enough spare time is available for engaging in PA, may be needed to increase PA levels in the Thai population [7].

It is interesting that consistent evidence of association with PA across all three age groups was found only for two psychological factors; namely self-efficacy and perceived barriers. Our finding regarding self-efficacy is in accordance with previous studies that identified this characteristic as an important determinant of PA behaviour across lifespan; from childhood to older age [2, 9, 25, 204, 205]. Furthermore, perceived barriers for PA are one’s evaluations of potential obstacles to start engaging in PA and/or to maintain regular PA (e.g. time constraints, lack of skills, unsuitable weather conditions). Although barriers for PA/exercise may be perceived differently by members of different age groups [206], they seem to be consistently negatively associated with PA behaviour for people of all ages in the Thai population. In previous, multinational reviews, including studies conducted in low-, middle-, and high-income countries, the authors reached inconsistent conclusions with respect to barriers to PA/exercise [2, 9, 24, 25]. For example, whilst Sallis et al. [24] suggested that perceived barriers are significantly associated with PA in children, van der Horst et al. suggested they are not [207]. Similarly, inconclusive findings for adults and older adults have also been reported by other authors [2, 25]. It may be that the association between perceived barriers to PA and PA levels is country-specific. Thus, acquiring country-level evidence may be needed to design effective interventions to tackle perceived barriers to PA.

Social support (e.g. from parents, family, and friends) was identified as a motivator for increased participation in PA, especially for Thai children/adolescents and adults. This is in accordance with findings of previous reviews [2, 9, 24, 208–210]. Interestingly, friend or peer influences have been shown to have a significant direct effect on PA among adolescents, while parents seem to have a more indirect influence [211]. In the current review, we found evidence suggesting that influences from both friends and parents may play important roles in children’s/adolescents’ engagement in PA. Several studies included in the present review also suggested that parental or family influences may be important correlates of PA in Thai adults. Although only one study showed a positive correlation between friendship/companionship influences and PA in this age group, the evidence should not be ignored, as this was a relatively large study [74]. Social support from friends and companions seem to be an enabler of PA also in Thai older adults. In this age group, support from hospital staff to engage in PA may be important. Therefore, improving different aspects of social support need to be taken into consideration when designing PA strategies and interventions for the Thai population.

Worldwide, a range of environmental attributes have been associated with PA, such as community resources, neighbourhood safety, transportation environment, access to sport and exercise facilities, routine destinations in daily life, and accessibility of public green spaces [2, 9, 18, 24, 25, 208–210, 212–216]. Interestingly, access and proximity to facilities appear to be important contributors to youth PA regardless of country’s economic status [2, 9, 24, 208, 209, 216]. Evidence on the associations between most aspects of physical environment and PA in Thai adults is inconclusive. This is mainly because of the limited number of studies conducted on this topic. Some evidence suggests that PA of children/adolescents and older adults in Thailand may be associated with neighbourhood design, which is consistent with findings from other, non-country specific reviews [217, 218]. Given the plethora of evidence from other countries and some evidence from Thailand, it seems important to improve features of the physical environment to increase PA participation in the Thai population. Nevertheless, more studies are needed to investigate environmental correlates of PA that are specific for Thailand.

In terms of policy-related correlates of PA, very few variables have been investigated. Only five studies have examined the effects of policies on PA in Thailand, and were carried out at the local level (i.e. the school and workplace) and among adults [70, 75, 84, 87, 219]. Some evidence suggests that education policies (in specific, university policies) may be associated with PA, whilst a single study did not find a significant association between workplace policies and PA. Further investigations on the potential impact of policies on PA is encouraged.

### Sedentary behaviour correlates

A limited number of studies have examined correlates of SB in Thai populations, especially in older adults. Some evidence suggests that, among Thai adults, males engage more in SB than females. This is in accordance with the associations between overall SB and sex found in several other countries [20]. Furthermore, consistent evidence from multiple studies was found for the association between obesity and SB in Thai adults. An umbrella review has suggested that available evidence is not supportive of this association in adults [220]. It might, therefore, be that the findings of the current review reflect only a context-specific situation in Thailand. Based on the available evidence, it seems that interventions for reducing SB should particularly focus on obese individuals, as being obese seems to be associated with more SB. It should be noted, however, that all Thai studies supporting this association are cross-sectional; hence, no inferences can be made about the direction of the relationship. Nevertheless, a previous longitudinal study suggested that obesity may lead to a subsequent increase in SB, whilst there was no evidence for the association in the other direction [221]. Besides, we found mostly non-significant associations between being overweight and engaging in SB. It might, therefore, be that only more severe issues with excessive weight lead to increases in SB. We also found some evidence supporting the association between SB and musculoskeletal disorders. Experiencing bodily pain was associated with higher SB in children and adolescents, whilst having musculoskeletal symptoms and low back pain were associated with higher SB in adults. These findings must be taken with caution, because previous longitudinal studies provided very little evidence in support of such associations [6, 222]. Furthermore, alcohol consumption was found to be associated with increased SB in both adults and older adults. These are, however, findings from one study only (examining both age groups), and given the inconsistent evidence for this association found in a previous review [20], this warrants further investigation. Associations of other variables and SB were either non-significant or supported by a single study, which demonstrates the need for more studies on SB in Thai populations, and particularly in older adults.

### Strengths and limitations

This systematic review has several strengths. Most previous reviews of PA/SB correlates did not present country-specific findings. In the current review, for the first time, findings on potential PA/SB correlates in the Thai population were extracted and summarised from many original studies (*n* = 167). Previous reviews on PA/SB correlates have cited primarily research published in the English language, which may have introduced bias into their findings. In this review, we included publications in both Thai and English; the languages that Thai researchers predominantly use in academic communication. Furthermore, we examined numerous potential correlates, particularly for PA, at all levels—individual, social, environmental, and policy. This comprehensive approach enabled us to better elucidate the complexity of PA and SB behaviours in the Thai population.

This review was not without limitations. Firstly, we did not use a formal meta-analytical procedure to combine the results of individual studies. Given the large number of analysed correlates and great heterogeneity between studies in terms of measures of PA/SB and statistical methods they used, we opted for the procedure for summarising results of individual studies proposed by Sallis et al. [24]. This procedure has been used in several previous systematic reviews in the field of public health. Secondly, relying on evidence from cross-sectional studies has prevented us from drawing conclusions about the direction of the summarised relationships. This was inevitable, because we did not identify any longitudinal studies on factors affecting PA/SB in Thailand. Finally, the findings of this review may have been influenced by recall errors, because the clear majority of included studies relied on self-reported PA/SB, and only a few used devices to assess these behaviours.

### Recommendations for future research

A limited number of studies examined SB correlates, particularly for older adults. More research is needed to understand why Thais engage in excessive SB and which factors to address to prevent it. Furthermore, less than one-fourth of all studies on PA correlates were conducted among children and adolescents. These age groups should, therefore, be designated as a priority target for future research on PA correlates. For most correlates of both PA and SB, only few findings from individual studies are available. This is particularly the case for social, environmental, and policy-related variables. More research is needed on most potential correlates of PA and SB in the Thai population. Another challenge stems from the fact that all studies included in this review used cross-sectional designs. To provide evidence on prospective and causal relationships between the variables and PA/SB, longitudinal and intervention studies should be conducted. In addition, to improve the validity of PA/SB estimates and avoid the potential recall bias, subject to feasibility, researchers are encouraged to employ devices, such as accelerometers, pedometers, or multi-sensor measures.

## Conclusions

This review is one of the first to summarise within-country correlates of PA and SB across population groups. Given a range of differences between the findings of the current review and the findings of previous non-country specific reviews, it may be important to consider correlates of PA and SB at the country-level. This may be particularly relevant when such reviews are completed to inform national- and local-level public health interventions.

Findings of the current review suggest that several factors are associated with PA levels in the Thai population. Based on the available evidence, to increase PA in Thailand, public health interventions should focus on helping individuals: improve self-efficacy; circumvent perceived barriers for PA; improve general and mental health; find enough spare time to engage in PA; improve physical skills, abilities, and fitness; gain knowledge about and experience in exercise; and receive adequate social support for participation in PA. Furthermore, the body of literature on correlates of SB in Thailand is limited. Nevertheless, evidence suggests that interventions for reducing SB in Thai adults should primarily target obese individuals, as they seem to be at a greater risk of high SB.

More Thai studies are needed on PA correlates, particularly among children and adolescent and with a focus on environment- and policy-related factors. Much greater commitment is needed to investigating correlates of SB in Thailand, particularly among older adults. The Thai Government and public health stakeholders should provide a systematic support to such research, as it provides knowledge that is crucial for designing public health policies, strategies, and interventions.

## Additional files

Additional file 1: Data Extraction Table. The detailed table of all data extracted from each study included. (XLSX 67 kb)

Additional file 2: Supplementary Correlate Tables. The additional tables of correlates for individual type of physical activity. (PDF 120 kb)

Additional file 3: Results of the study quality assessment using the Newcastle-Ottawa Scale for cross-sectional studies. The quality assessment score for included studies assessed by Newcastle-Ottawa Scale (NOS). (PDF 349 kb)

## Abbreviations

MPA: Moderate Physical Activity
MVPA: Moderate-to-Vigorous Physical Activity
NDLTD: Networked Digital Library of Theses and Dissertations
PA: Physical Activity
SB: Sedentary Behaviour
TV: Television
VPA: Vigorous Physical Activity
WHO: World Health Organization
