# Supplementary material for: Correlates of physical activity and sedentary behaviour in the Thai population: a systematic review
Source: BMC Public Health. 2019 Apr 16;19:414. doi: 10.1186/s12889-019-6708-2 (PMC6469108; doi:10.1186/s12889-019-6708-2)
Supplement: Supplementary file 2 — Supplementary Correlate Tables. The additional tables of correlates for individual type of physical activity. (PDF 120 kb) [file 12889_2019_6708_MOESM2_ESM.pdf]

Supplementary Table 1: Summary of evidence on moderate-to-vigorous physical activity (MVPA) in Thai children and adolescents (6 – 17 years old)

Correlates	Relationship with PA			Summary Code
	Negative/Inverse (-)	Positive (+)	Non-significant (?)	
Individual Level				
Demographic factors				
– Age	97			-
– Sex (+ denotes males are more active, - denotes females are more active)		97, 126, 135		+
– Household income	96			-
– Municipality (+ denotes more PA in an urban place of residence, - denotes more PA in a rural place of residence)			135 (M, F)	?
Biological factors				
– Body mass index (BMI)	97, 130 (M)		130 (F)	-
General health				
– Child's health status as perceived by parents			45	?
Psychological, cognitive, and emotional factors				
– Self-efficacy		45		+
– Perceived benefits of PA/exercise			38 (M, F)	?
– Perceived barriers for PA/exercise	38 (F)		38 (M)	?
– Cues to action			38 (M, F)	?
– Being bullied	129 (M)		129 (F)	?
Knowledge				
– Parents' knowledge about school-aged children's PA			45	?

(+) Mostly positive associations, (-) Mostly negative associations, (?) Mostly non-significant, indeterminate, or inconsistent associations, (M) Male, (F) Female

Supplementary Table 2: Summary of evidence on total physical activity in Thai children and adolescents (6 – 17 years old)

Correlates	Relationship with PA			Summary Code
	Negative/Inverse (-)	Positive (+)	Non-significant (?)	
Individual Level				
Demographic factors				
– Age	135 (M, F), 183		158 (M, F)	-
– Sex (+ denotes males are more active, - denotes females are more active)		55, 135, 158, 178, 183	169	++
– School type (+ denotes more PA in mixed-gender schools, - denotes more PA in single-gender schools)		55		+
– Household income	178			
– Municipality (+ denotes more PA in an urban place of residence, - denotes more PA in a rural place of residence)		169	135 (M, F)	?
– Household's location within Bangkok			178	?
Biological factors				
– Body weight			135 (M, F)	?
– Body mass index (BMI)	178		135 (M, F), 158	?
– Parents' BMI			178	?
– Overweight			52	?
– Obesity			169	?
– Body fat (%)		135 (M)	135 (F)	?
– Height	135 (M, F)			-
– Low-density lipoprotein cholesterol (LDL-C) level			158	?
– Systolic blood pressure			158	?
– Diastolic blood pressure			158	?
– Dietary fat intake			158	?
Psychological, cognitive, and emotional factors				
– Attitudes towards PA/exercise		178		+
– Body dissatisfaction	55			-
– Enjoyment of PA/exercise		183	178	?

Correlates	Relationship with PA			Summary Code
	Negative/Inverse (-)	Positive (+)	Non-significant (?)	
Physical skills, abilities, and fitness				
– Perceived physical competence		183		+
Health behavioural and lifestyle				
– TV viewing	178			-
Social environment				
Social and cultural factors				
– Social support		178		+
– Parent/family influences		183	178	?
– Friendship/companionship influences		183		+
Physical environment				
Environment factors				
– Environmental support (e.g. situational influences)		178		+
– Supportive physical environment (e.g. facilities, supplies)		178		+
– Supportive neighbourhood environment		178		+

(+) Mostly positive associations, (-) Mostly negative associations, (?) Mostly non-significant, indeterminate, or inconsistent associations, (M) Male, (F) Female

Supplementary Table 3: Summary of evidence on moderate physical activity (MPA) in Thai children and adolescents (6 – 17 years old)

Correlates	Relationship with PA			Summary Code
	Negative/Inverse (-)	Positive (+)	Non-significant (?)	
Individual Level				
Demographic factors				
– Sex (+ denotes males are more active, - denotes females are more active)		111, 118		+
– School grade			118	?
Biological factors				
– Body mass index (BMI)			33	?
– Overweight		111 (F)	111 (M)	?
Psychological, cognitive, and emotional factors				
– Resilience		118		+

(+) Mostly positive associations, (-) Mostly negative associations, (?) Mostly non-significant, indeterminate, or inconsistent associations, (M) Male, (F) Female

Supplementary Table 4: Summary of evidence on vigorous physical activity (VPA) in Thai children and adolescents (6 – 17 years old)

Correlates	Relationship with PA			Summary Code
	Negative/Inverse (-)	Positive (+)	Non-significant (?)	
Individual Level				
Demographic factors				
– Sex (+ denotes males are more active, - denotes females are more active)		118, 122		+
– School grade			118	?
General health				
– Self-rated general health		121 (M, F)		+
Psychological, cognitive, and emotional factors				
– Resilience		118		+
Social environment				
Social and cultural factors				
– Friendship/companionship influences		122 (M, F)		+
– Involvement with friends		122 (M, F)		+
– Ease in making friends		122 (M, F)		+

(+) Mostly positive associations, (-) Mostly negative associations, (?) Mostly non-significant, indeterminate, or inconsistent associations, (M) Male, (F) Female

Supplementary Table 5: Summary of evidence on exercise in Thai children and adolescents (6 – 17 years old)

Correlates	Relationship with PA			Summary Code
	Negative/Inverse (-)	Positive (+)	Non-significant (?)	
Individual Level				
Demographic factors				
– Sex (+ denotes males are more active, - denotes females are more active)		51, 167	189	+
– School grade	80		51, 167, 189	?
– Parents' occupation			189	?
– Municipality (+ denotes more PA in an urban place of residence, - denotes more PA in a rural place of residence)			64	?
Biological factors				
– Body mass index (BMI)			51	?
– Underweight			110	?
– Overweight	110, 157		125 (F)	-
– Obesity	110, 157		125 (F)	-
– Height			80 (M, F)	?
Physical health				
– Asthma	157			-
– Hypertension	157			-
Psychological, cognitive, and emotional factors				
– Self-efficacy		51, 80, 123	167	+
– Perceived benefits of PA/exercise		48, 77	167	+
– Perceived barriers for PA/exercise	51, 80, 123		167	-
– Attitudes towards PA/exercise			167	?
– Self-esteem			167	?
– Expected outcomes of PA/exercise			167	?
– Enjoyment of PA/exercise		80		+
Health behavioural and lifestyle				

Correlates	Relationship with PA			Summary Code
	Negative/Inverse (-)	Positive (+)	Non-significant (?)	
– Past PA/exercise experience		51, 80	167	+
Knowledge				
– Knowledge about PA/exercise		167		+
Academic/school performance				
– Grade point average	157			-
Social environment				
Social and cultural factors				
– Social support		123	167	?
– Parent/family influences		80		+
– Friendship/companionship influences		80		+
– Teacher influences		80		+
– General interpersonal influences		51		+
– Information support (e.g. social media)		167		+
Physical environment				
Environment factors				
– Environmental support (e.g. situational influences)		51, 80		+
– Supportive physical environment (e.g. facilities, supplies)			167	?

(+) Mostly positive associations, (-) Mostly negative associations, (?) Mostly non-significant, indeterminate, or inconsistent associations, (M) Male, (F) Female

Supplementary Table 6: Summary of evidence on moderate-to-vigorous physical activity (MVPA) in Thai adults (18 – 59 years old)

Correlates	Relationship with PA			Summary Code
	Negative/Inverse (-)	Positive (+)	Non-significant (?)	
Individual Level				
Demographic factors				
– Age			116	?
– Sex (+ denotes males are more active, - denotes females are more active)		31, 116, 131, 132, 136, 180, 181		++
– Marital status (+denotes singles are more active)			101	?
– Education level	116		101	?
– Household income			101	?
– Occupation (+denotes unemployed are more active)			101	?
– Early-life (0-5) urban exposure			35	?
– Number of family members			101	?
– Student residency (+ denotes residents of university dorms are more active, - denotes students living in other accommodation types are more active)			181	?
Biological factors				
– Body mass index (BMI)		180	181	?
– Underweight		131 (F)	131 (M)	?
– Overweight			131 (M, F), 146 (M, F)	??
– Obesity			128, 131 (M, F)	?
– Total cholesterol (TCH) level			60 (M, F)	?
– Triglyceride (TG) level	60 (M, F)			-
– HDL-Cholesterol level		60 (M, F)		+
– Total cholesterol: HDL-C ratio	60 (M, F)			-
– High TCH			60 (M, F)	?
– High TG	60 (M, F)			-
– Low HDL-C			60 (M, F)	?
– High TCH: HDL-C ratio			60 (M, F)	?

Correlates	Relationship with PA			Summary Code
	Negative/Inverse (-)	Positive (+)	Non-significant (?)	
– Resting heart rate	166 (M, F)			-
– Mean heart rate			166 (M, F)	?
– Predicted maximum heart rate (%)			166 (M, F)	?
– Systolic blood pressure			166 (M, F)	?
– Diastolic blood pressure			166 (M, F)	?
– VO_2max		166 (M, F)		+
– FEV1 (Abnormal lung function)			166 (M, F)	?
– FVC (Pulmonary function test)			166 (M, F)	?
– FEV1/FVC (%)			166 (M, F)	?
Physical health				
– History of sickness/Underlying illness/Co-morbid diseases (+ denotes yes, - denotes no)			101, 181	?
– Metabolic syndrome			136 (M, F)	?
– Musculoskeletal symptoms			89	?
– Relative appendicular skeletal muscle mass (RASM)		105 (F)	105 (M)	?
Psychological, cognitive, and emotional factors				
– Self-efficacy			101	?
– Perceived benefits of PA/exercise		101		+
– Perceived barriers for PA/exercise			101	?
– Sense of coherence			108	?
– Identity achievement			108	?
Physical skills, abilities, and fitness				
– Physical and functional fitness (e.g. walking distance, leg strength)		166 (M, F)		+
Health behavioural and lifestyle				
– Smoking			78 (M, F), 153	?
– Sedentary time			127	?
– Monosodium glutamate (MSG) intake			71	?

Correlates	Relationship with PA			Summary Code
	Negative/Inverse (-)	Positive (+)	Non-significant (?)	
Knowledge				
– Knowledge about PA/exercise		101		+
Social environment				
Social and cultural factors				
– Social support			101	?
– Cultural support		180		+
Physical environment				
Environment factors				
– Supportive physical environment (e.g. facilities, supplies)			101	?
– Urban environment	35			-

(+) Mostly positive associations, (-) Mostly negative associations, (?) Mostly non-significant, indeterminate, or inconsistent associations, (M) Male, (F) Female

Supplementary Table 7: Summary of evidence on total physical activity in Thai adults (18 – 59 years old)

Correlates	Relationship with PA			Summary Code
	Negative/Inverse (-)	Positive (+)	Non-significant (?)	
Individual Level				
Biological factors				
– Body mass index (BMI)			109	?
– Overweight			114	?
– Obesity	86, 114			-
– Body fat (%)			109	?
– Bone mineral density (BMD)		138		+
– Dietary calcium intake		138		+
– Sunlight exposure		138		+
Physical health				
– Osteoporosis		138		+
Psychological, cognitive, and emotional factors				
– Attitudes towards PA/exercise		154		+
Knowledge				
– Knowledge about PA	154			-
Social environment				
Social and cultural factors				
– Social support		154		+
– General interpersonal influences		154		+
– Information support (e.g. from media)		154		+
Physical environment				
Environment factors				
– Supportive physical environment (e.g. facilities, supplies)		154		+
– Month of the year (+ denotes higher PA in later months of a calendar year)		109		+
Policy				
Policy attributes				

Correlates	Relationship with PA			Summary Code
	Negative/Inverse (-)	Positive (+)	Non-significant (?)	
– Supportive education policies		154		+

(+) Mostly positive associations, (-) Mostly negative associations, (?) Mostly non-significant, indeterminate, or inconsistent associations, (M) Male, (F) Female

Supplementary Table 8: Summary of evidence on vigorous physical activity (VPA) in Thai adults (18 – 59 years old)

Correlates	Relationship with PA			Summary Code
	Negative/Inverse (-)	Positive (+)	Non-significant (?)	
Individual Level				
Demographic factors				
– Sex (+ denotes males are more active, - denotes females are more active)		184		+
Social environment				
Social and cultural factors				
– Religion (+ denotes Buddhists are more active, - denotes Muslims are more active)		184		+

(+) Mostly positive associations, (-) Mostly negative associations, (?) Mostly non-significant, indeterminate, or inconsistent associations, (M) Male, (F) Female

Supplementary Table 9: Summary of evidence on exercise in Thai adults (18 – 59 years old)

Correlates	Relationship with PA			Summary Code
	Negative/Inverse (-)	Positive (+)	Non-significant (?)	
Individual Level				
Demographic factors				
– Age		37, 59, 107, 124, 140, 172	72, 81, 112, 117, 155, 176, 190, 197	??
– Sex (+ denotes males are more active, - denotes females are more active)	50, 100, 155, 196	83, 87, 100, 107, 113, 117, 150, 159, 165, 186, 190, 193, 195	36, 44, 59, 72, 92, 95, 112, 124, 140, 172, 176, 194, 197	??
– Marital status (+denotes singles are more active)		155	59, 72, 81, 112, 117, 172, 190, 197	??
– Education level	117	59, 155, 190	72, 112, 172, 197	??
– University year	165	137, 186	36, 95, 179, 193, 195	??
– Faculty*		44, 87, 165, 194, 195, 196	92, 95, 159, 179, 186	??
– Household income	117	92	59, 72, 172, 179, 190, 195, 197	??
– Occupation (+denotes unemployed are more active)		155, 190	59, 117	?
– Region (+denotes Central including Bangkok residents are more active, -denotes residents of other provinces are more active)	134	155	172	?
– Student residency (+ denotes residents of university dorms are more active, -denotes students living in other accommodation types are more active)		195	95	?
– Hometown (urban/rural)			92	?
– Campus/working location*		163, 197	44, 112, 159, 194	??
– Having a dependant			81	?
– Years of working experience		172		+
– Working position			112, 172, 197	?
– Working type			197	?

Correlates	Relationship with PA			Summary Code
	Negative/Inverse (-)	Positive (+)	Non-significant (?)	
– Duration of health club membership			117	?
– Having a ‘dream job’		92		+
– Extra job (+ denotes yes, - denotes no)			112	?
– Ever attended a workshop on exercise (+ denotes yes, - denotes no)		124		+
Biological factors				
– Body mass index (BMI)	179		197	?
– Underweight		150		+
– Overweight	150		65 (M, F)	?
– Obesity	46 (M, F), 47 (M, F), 150		65 (M, F)	--
– Waist circumference			197	?
– Bone mineral density (BMD)		134	74, 90, 145	?
– Skeletal muscle mass percent			143	?
– Adequate serum vitamin D levels			143	?
– Total cholesterol (TCH) level			107	?
– Triglyceride (TG) level	107			-
– HDL-Cholesterol level		107		+
– LDL-Cholesterol level			107	?
– Resting heart rate	107			-
– Systolic blood pressure			107	?
– Diastolic blood pressure			107	?
– Fasting plasma glucose			107	?
– Hematocrit level	73			-
– Hypercholesterolemia			103 (M, F)	?
Physical health				
– History of sickness/Underlying illness/Co-morbid diseases (+ denotes yes, - denotes no)			59, 112, 195, 197	??
– Hypertension			69	?

Correlates	Relationship with PA			Summary Code
	Negative/Inverse (-)	Positive (+)	Non-significant (?)	
– Musculoskeletal symptoms	63, 144		85, 133	?
– Dysmenorrhea			57 (F)	?
– Age-related macular degeneration (AMD)	88			-
General health				
– Self-rated general health		87, 94, 175, 194, 197	159	++
Psychological, cognitive, and emotional factors				
– Mental health		32, 94, 175, 190		++
– Attitudes towards PA/exercise		49, 50 (M, F), 117, 165, 186, 188, 190, 197	44, 70, 82, 112	++
– Overall health belief		170		+
– Self-efficacy		81, 83, 87, 140, 159, 172, 194	72, 137	++
– Perceived benefits of PA/exercise		50 (M, F), 83, 87, 137, 172, 175, 194	59, 72, 159, 170	++
– Perceived barriers for PA/exercise	72, 83, 87, 159, 170, 172, 175, 194		62	--
– Outcome expectancies		81, 140		+
– Intention to PA/exercise		140, 179		+
– Enjoyment of PA/exercise		59		+
– Perceived exercise-related effect	140		72, 170	?
– Career satisfaction		182		+
– Motivation for PA/exercise		83	76, 195	?
– External regulation			76	?
– Extrinsic motivation	76			-
– Intrinsic motivation		76		+
– Commitment to PA/exercise			151	?
– Stress level	179			-

Correlates	Relationship with PA			Summary Code
	Negative/Inverse (-)	Positive (+)	Non-significant (?)	
– Score on memory and intelligence tests			32	?
Physical skills, abilities, and fitness				
– Physical and functional fitness (e.g. walking distance, leg strength)		94		+
Health behavioural and lifestyle				
– Alcohol consumption		177 (M)	177 (F)	?
– Healthy dietary habits		119		+
– Past PA/exercise experience		72		+
– Being a university athlete			137, 165	?
– Availability and/or engagement in enjoyable sports		115, 137		+
– Loving watching sports			115	?
– Having a favourite athlete			115	?
– Spare time		49, 59, 82, 115		++
Knowledge				
– Knowledge about PA		44, 50 (M, F), 59, 117, 188, 194	49, 82, 87, 112, 159, 165, 195, 197	?
Academic/school performance				
– Grade point average			179	?
Social environment				
Social and cultural factors				
– Social support		50 (M, F), 87, 159, 165, 194, 197		++
– General interpersonal influences	151	49, 82, 197	117	+
– Parent/family influences		81, 83, 137, 172	72, 117	++
– Family members’ involvement in PA/exercise		137		+
– Friendship/companionship influences		81		+
– Friends’ involvement in PA/exercise			115	?
– Information support (e.g. from media)		49, 159, 194, 197	82	++

Correlates	Relationship with PA			Summary Code
	Negative/Inverse (-)	Positive (+)	Non-significant (?)	
– Religion (+ denotes Buddhists are more active, - denotes Muslims are more active)			95	?
Physical environment				
Environment factors				
– Environmental support (e.g. situational influences)		137	72, 151, 195	?
– Supportive physical environment (e.g. facilities, supplies)		81, 82, 87	49, 197	+
– Supportive neighbourhood environment			81	?
– Urban environment	100 (M, F)			-
– Convenience of travel			115	?
Policy				
Policy attributes				
– Supportive education policies		82, 165	49	+
– Supportive workplace policies			197	?

(+) Mostly positive associations, (-) Mostly negative associations, (?) Mostly non-significant, indeterminate, or inconsistent associations, (M) Male, (F) Female, Faculty* and Campus/working location* variables - due to a number of categories of these variables, we used only + (to denote any significant association of specific faculties or campuses/working locations) and ? (to denote a non-significant association) codes.

Supplementary Table 10: Summary of evidence on occupation physical activity in Thai adults (18 – 59 years old)

Correlates	Relationship with PA			Summary Code
	Negative/Inverse (-)	Positive (+)	Non-significant (?)	
Individual Level				
Biological factors				
– Bone mineral density (BMD)		160 (M)	160 (F), 160 (M, F)	?
– Calcaneal stiffness index			160 (M, F)	?

(+) Mostly positive associations, (-) Mostly negative associations, (?) Mostly non-significant, indeterminate, or inconsistent associations, (M) Male, (F) Female

Supplementary Table 11: Summary of evidence on transportation physical activity in Thai adults (18 – 59 years old)

Correlates	Relationship with PA			Summary Code
	Negative/Inverse (-)	Positive (+)	Non-significant (?)	
Individual Level				
Demographic factors				
– Age		58		+
– Sex (+ denotes males are more active, - denotes females are more active)			58	?
– Municipality (+ denotes more PA in an urban place of residence, - denotes more PA in a rural place of residence)			58	?
– Marital status (+ denotes singles are more active)			58	?
– Education level	58			-
– Household income			58	?
– Occupation (+ denotes unemployed are more active)			58	?
Biological factors				
– Body mass index (BMI)			58	?
– Bone mineral density (BMD)		160 (M)	160 (M, F)	?
– Calcaneal stiffness index			160 (M, F)	?
Health behavioural and lifestyle				
– Sedentary time	58			-
Physical environment				
Environment factors				
– Distance to work			58	?
– Distance to the nearest shopping place	58			-
– Distance to the nearest religious establishment			58	?

(+) Mostly positive associations, (-) Mostly negative associations, (?) Mostly non-significant, indeterminate, or inconsistent associations, (M) Male, (F) Female

Supplementary Table 12: Summary of evidence on recreation physical activity in Thai adults (18 – 59 years old)

Correlates	Relationship with PA			Summary Code
	Negative/Inverse (-)	Positive (+)	Non-significant (?)	
Individual Level				
Demographic factors				
– Age			58	?
– Sex (+ denotes males are more active, - denotes females are more active)			58	?
– Municipality (+ denotes more PA in an urban place of residence, - denotes more PA in a rural place of residence)	58			-
– Marital status (+ denotes singles are more active)			58	?
– Education level			58	?
– Household income		58		+
– Occupation (+ denotes unemployed are more active)			58	?
Biological factors				
– Body mass index (BMI)			58	?
– Bone mineral density (BMD)		160 (M, F)		+
– Calcaneal stiffness index		160 (M, F)		+
– Hypercholesterolemia			103 (M, F)	?
Psychological, cognitive, and emotional factors				
– Self-efficacy		187		+
– Perceived benefits of PA/exercise			187	?
– Perceived barriers for PA/exercise			187	?
Health behavioural and lifestyle				
– Sedentary time	58			-
Social environment				
Social and cultural factors				
– General interpersonal influences		187		+
Physical environment				
Environment factors				

Correlates	Relationship with PA			Summary Code
	Negative/Inverse (-)	Positive (+)	Non-significant (?)	
– Distance to the nearest recreation facility			58	?

(+) Mostly positive associations, (-) Mostly negative associations, (?) Mostly non-significant, indeterminate, or inconsistent associations, (M) Male, (F) Female

Supplementary Table 13: Summary of evidence on domestic physical activity in Thai adults (18 – 59 years old)

Correlates	Relationship with PA			Summary Code
	Negative/Inverse (-)	Positive (+)	Non-significant (?)	
Individual Level				
Demographic factors				
– Sex (+ denotes males are more active, - denotes females are more active)	100			-
Biological factors				
– Obesity	46 (M, F), 47 (M, F)			--
Physical environment				
Environment factors				
– Urban environment	100 (M, F)			-

(+) Mostly positive associations, (-) Mostly negative associations, (?) Mostly non-significant, indeterminate, or inconsistent associations, (M) Male, (F) Female

Supplementary Table 14: Summary of evidence on moderate-to-vigorous physical activity (MVPA) in Thai older adults (60 years old and above)

Correlates	Relationship with PA			Summary Code
	Negative/Inverse (-)	Positive (+)	Non-significant (?)	
Individual Level				
Demographic factors				
– Age	104		66, 116, 142	?
– Sex (+ denotes males are more active, - denotes females are more active)	66	31, 116, 142		+
– Marital status (+ denotes singles are more active)		66		+
– Education level	116	142	66	?
– Household income	66		142	?
– Senior-citizen club membership (+ denotes yes, - denotes no)			66	?
Biological factors				
– Body mass index (BMI)			66	?
– Overweight			146 (M, F)	?
– Total cholesterol (TCH) level			60 (M, F)	?
– Triglyceride (TG) level	60 (M, F)			-
– HDL-Cholesterol level		60 (M, F)		+
– Total cholesterol: HDL-C ratio	60 (M, F)			-
– High TCH			60 (M, F)	?
– High TG	60 (M, F)			-
– Low HDL-C			60 (M, F)	?
– High TCH: HDL-C ratio			60 (M, F)	?
– Resting heart rate	166 (M, F)			-
– Mean heart rate			166 (M, F)	?
– Predicted maximum heart rate (%)			166 (M, F)	?
– Systolic blood pressure			166 (M, F)	?
– Diastolic blood pressure			166 (M, F)	?
– VO_2max		166 (M, F)		+

Correlates	Relationship with PA			Summary Code
	Negative/Inverse (-)	Positive (+)	Non-significant (?)	
– FEV1 (Abnormal lung function)			166 (M, F)	?
– FVC (Pulmonary function test)			166 (M, F)	?
– FEV1/FVC (%)			166 (M, F)	?
Physical health				
– History of sickness/Underlying illness/Co-morbid diseases (+ denotes yes, - denotes no)	66			-
Psychological, cognitive, and emotional factors				
– Self-efficacy		104		+
– Outcome expectancies		104		+
Physical skills, abilities, and fitness				
– Physical and functional fitness (e.g. walking distance, leg strength)		166 (M, F)		+
Health behavioural and lifestyle				
– Smoking	66		78 (M, F), 153	?
– Alcohol consumption	66			-
Social environment				
Social and cultural factors				
– Parent/family influences			104	?
– Friendship/companionship influences			104	?
Physical environment				
Environment factors				
– Supportive neighbourhood environment		104		+

(+) Mostly positive associations, (-) Mostly negative associations, (?) Mostly non-significant, indeterminate, or inconsistent associations, (M) Male, (F) Female

Supplementary Table 15: Summary of evidence on total physical activity in Thai older adults (60 years old and above)

Correlates	Relationship with PA			Summary Code
	Negative/Inverse (-)	Positive (+)	Non-significant (?)	
Individual Level				
Demographic factors				
– Age	139, 164			-
– Sex (+ denotes males are more active, - denotes females are more active)		152	39, 68, 139, 164	??
– Marital status (+ denotes singles are more active)			139, 164	?
– Education level		40, 139, 164	39	+
– Household income		39, 40, 139		+
– Senior-citizen club membership (+ denotes yes, - denotes no)		139		+
Biological factors				
– Bone mineral density (BMD)		99, 138		+
– Dietary calcium intake		138		+
– Sunlight exposure		138		+
Physical health				
– Osteoporosis	138			-
General health				
– Self-rated general health		98, 139, 164		+
– Health-related quality of life		56		+
– General oral-health status (i.e. number of teeth, and oral malodour)			152	?
– Periodontal disease	152			-
– Saliva flow rate		152		+
Psychological, cognitive, and emotional factors				
– Mental health		98		+
– Self-efficacy		98, 139	39, 53	?
– Perceived benefits of PA/exercise		40, 139	39	+
– Perceived barriers for PA/exercise	39, 40, 139			-

Correlates	Relationship with PA			Summary Code
	Negative/Inverse (-)	Positive (+)	Non-significant (?)	
– Commitment to an exercise plan		53		+
Health behavioural and lifestyle				
– Oral health behaviours (i.e. tooth brushing, and regular dental visits)		152		+
Social environment				
Social and cultural factors				
– Social support		53	98	?
– General interpersonal influences		139	117	?
– Parent/family influences			39	?
– Information support (e.g. from media)		164		+
Physical environment				
Environment factors				
– Supportive physical environment (e.g. facilities, supplies)		39		+
– Supportive neighbourhood environment		39, 40	98	+
– A sense of community		98		+
– Residential community (+ denotes rural community is more active, - denotes residential home is more active)		78		+

(+) Mostly positive associations, (-) Mostly negative associations, (?) Mostly non-significant, indeterminate, or inconsistent associations, (M) Male, (F) Female

Supplementary Table 16: Summary of evidence on moderate physical activity (MPA) in Thai older adults (60 years old and above)

Correlates	Relationship with PA			Summary Code
	Negative/Inverse (-)	Positive (+)	Non-significant (?)	
Individual Level				
Psychological, cognitive, and emotional factors				
– Mental health			43	?
Physical skills, abilities, and fitness				
– Physical and functional fitness (e.g. walking distance, leg strength)		43		+

(+) Mostly positive associations, (-) Mostly negative associations, (?) Mostly non-significant, indeterminate, or inconsistent associations, (M) Male, (F) Female

Supplementary Table 17: Summary of evidence on vigorous physical activity (VPA) in Thai older adults (60 years old and above)

Correlates	Relationship with PA			Summary Code
	Negative/Inverse (-)	Positive (+)	Non-significant (?)	
Individual Level				
Demographic factors				
– Sex (+ denotes males are more active, - denotes females are more active)		184		+
Psychological, cognitive, and emotional factors				
– Mental health		43		+
Physical skills, abilities, and fitness				
– Physical and functional fitness (e.g. walking distance, leg strength)		43		+
Social environment				
Social and cultural factors				
– Religion (+ denotes Buddhists are more active, - denotes Muslims are more active)		184		+

(+) Mostly positive associations, (-) Mostly negative associations, (?) Mostly non-significant, indeterminate, or inconsistent associations, (M) Male, (F) Female

Supplementary Table 18: Summary of evidence on transportation physical activity in Thai older adults (60 years old and above)

Correlates	Relationship with PA			Summary Code
	Negative/Inverse (-)	Positive (+)	Non-significant (?)	
Individual Level				
Demographic factors				
– Age		58		+
– Sex (+ denotes males are more active, - denotes females are more active)			58	?
– Municipality (+ denotes more PA in an urban place of residence, - denotes more PA in a rural place of residence)			58	?
– Marital status (+ denotes singles are more active)			58	?
– Education level	58			-
– Household income			58	?
– Occupation (+ denotes unemployed are more active)			58	?
Biological factors				
– Body mass index (BMI)			58	?
Health behavioural and lifestyle				
– Sedentary time	58			-
Physical environment				
Environment factors				
– Distance to work			58	?
– Distance to the nearest shopping place	58			-
– Distance to the nearest religious establishment			58	?

(+) Mostly positive associations, (-) Mostly negative associations, (?) Mostly non-significant, indeterminate, or inconsistent associations, (M) Male, (F) Female

Supplementary Table 19: Summary of evidence on recreation physical activity in Thai older adults (60 years old and above)

Correlates	Relationship with PA			Summary Code
	Negative/Inverse (-)	Positive (+)	Non-significant (?)	
Individual Level				
Demographic factors				
– Age			58	?
– Sex (+ denotes males are more active, - denotes females are more active)			58	?
– Municipality (+ denotes more PA in an urban place of residence, - denotes more PA in a rural place of residence)	58			-
– Marital status (+ denotes singles are more active)			58	?
– Education level			58	?
– Household income		58		+
– Occupation (+ denotes unemployed are more active)			58	?
Biological factors				
– Body mass index (BMI)			58	?
– Hypercholesterolemia			103 (M, F)	?
General health				
– Health-related quality of life		48		+
Health behavioural and lifestyle				
– Sedentary time	58			-
Physical environment				
Environment factors				
– Distance to the nearest recreation facility			58	?

(+) Mostly positive associations, (-) Mostly negative associations, (?) Mostly non-significant, indeterminate, or inconsistent associations, (M) Male, (F) Female

Supplementary Table 20: Summary of evidence on occupation physical activity in Thai older adults (60 years old and above)

Correlates	Relationship with PA			Summary Code
	Negative/Inverse (-)	Positive (+)	Non-significant (?)	
Individual Level				
General health				
– Health-related quality of life		48		+

(+) Mostly positive associations, (-) Mostly negative associations, (?) Mostly non-significant, indeterminate, or inconsistent associations, (M) Male, (F) Female

Supplementary Table 21: Summary of evidence on domestic physical activity in Thai older adults (60 years old and above)

Correlates	Relationship with PA			Summary Code
	Negative/Inverse (-)	Positive (+)	Non-significant (?)	
Individual Level				
Demographic factors				
– Sex (+ denotes males are more active, - denotes females are more active)	100			-
Biological factors				
– Obesity	47 (M, F)			-
General health				
– Health-related quality of life		48		+
Physical environment				
Environment factors				
– Urban environment	100 (M, F)			-

(+) Mostly positive associations, (-) Mostly negative associations, (?) Mostly non-significant, indeterminate, or inconsistent associations, (M) Male, (F) Female

Supplementary Table 22: Summary of evidence on exercise in Thai older adults (60 years old and above)

Correlates	Relationship with PA			Summary Code
	Negative/Inverse (-)	Positive (+)	Non-significant (?)	
Individual Level				
Demographic factors				
– Age	191, 192	37, 59, 124, 140	117, 155, 162, 174, 176, 185	??
– Sex (+ denotes males are more active, - denotes females are more active)	155	42, 100, 117, 162, 174, 185	59, 124, 140, 176, 191	??
– Municipality (+ denotes more PA in an urban place of residence, - denotes more PA in a rural place of residence)		192		+
– Marital status (+ denotes singles are more active)		155	59, 117, 174, 185, 191	??
– Education level	117	59, 155, 162, 185, 192	174, 191	++
– Household income	117	185, 182	59, 162	?
– Occupation (+ denotes unemployed are more active)		155	59, 117	?
– Region (+ denotes Central including Bangkok residents are more active, - denotes residents of other provinces are more active)	134	155		?
– Daily duties (+ denotes individuals performing daily duties regularly are more active, - denotes individuals performing daily duties occasionally are more active)	162			-
– Senior-citizen club membership (+ denotes yes, - denotes no)		191		+
– Duration of health club membership			117	?
– Ever attended a workshop on exercise (+ denotes yes, - denotes no)		124		+
Biological factors				
– Obesity	47 (M, F)			-
– Bone mineral density (BMD)	42 (F)	134	42 (M)	?
– Skeletal muscle mass (%)			143	?
– Adequate serum vitamin D levels			143	?
– Systolic blood pressure	161			-
– Diastolic blood pressure	161			-

Correlates	Relationship with PA			Summary Code
	Negative/Inverse (-)	Positive (+)	Non-significant (?)	
– Hematocrit level	73			-
– Hypercholesterolemia			103 (M, F)	?
Physical health				
– History of sickness/Underlying illness/Co-morbid diseases (+ denotes yes, - denotes no)			59, 162	?
– Having health symptoms (i.e. fatigue, weight loss, sleep disorders) (+ denotes yes, - denotes no)	162			-
– Osteoporosis				
– Age-related macular degeneration (AMD)	88			-
General health				
– Self-rated general health		168, 175	185, 192	?
– Health-related quality of life		41		+
Psychological, cognitive, and emotional factors				
– Mental health		32, 175		+
– Attitudes towards PA/exercise		79, 117, 188, 191		++
– Self-efficacy		79, 140, 162, 185	174	++
– Perceived benefits of PA/exercise		79, 174, 176, 192	59	++
– Perceived barriers for PA/exercise	79, 176		174	-
– Outcome expectancies		79, 140, 162		+
– Intension to PA/exercise		79, 140		+
– Perceived control		79		+
– Subjective norms		79		+
– Enjoyment of PA/exercise		59		+
– Perceived health risks of exercise	140			-
– Life satisfaction		120		+
– Career satisfaction		182		+
– Motivation for PA/exercise			174	?

Correlates	Relationship with PA			Summary Code
	Negative/Inverse (-)	Positive (+)	Non-significant (?)	
– Score on memory and intelligence tests			32	?
Physical skills, abilities, and fitness				
– Ability to do daily activities		192		+
– Physical and functional fitness (e.g. walking distance, leg strength)		34, 93		+
– Score on Timed Up and Go test	93			-
Health behavioural and lifestyle				
– Alcohol consumption		177 (M)	177 (F)	?
– Spare time		59		+
Knowledge				
– Knowledge about PA/exercise		59, 117, 188, 191	162	++
Social environment				
Social and cultural factors				
– Social support		192	162, 174	?
– General interpersonal influences			117	?
– Parent/family influences		191, 192		+
– Friendship/companionship influences		191, 192		+
– Hospital staff support		191		+
– Information support (e.g. from media)				
– Religion (+ denotes Buddhists are more active, - denotes Muslims are more active)		192		+
Physical environment				
Environment factors				
– Supportive physical environment (e.g. facilities, supplies)		192	117	?
– Urban environment	100 (M, F)			-

(+) Mostly positive associations, (-) Mostly negative associations, (?) Mostly non-significant, indeterminate, or inconsistent associations, (M) Male, (F) Female
