# Supplementary material for: Correlates of physical activity and sedentary behaviour in the Thai population: a systematic review
Source: BMC Public Health. 2019 Apr 16;19:414. doi: 10.1186/s12889-019-6708-2 (PMC6469108; doi:10.1186/s12889-019-6708-2)
Supplement: Supplementary file 3 — Results of the study quality assessment using the Newcastle-Ottawa Scale for cross-sectional studies. The quality assessment score for included studies assessed by Newcastle-Ottawa Scale (NOS). (PDF 349 kb) [file 12889_2019_6708_MOESM3_ESM.pdf]

Results of the study quality assessment using the Newcastle-Ottawa Scale for cross-sectional studies

No.	Item	Sample representativeness	Sample size	Non-respondents	Ascertainment of exposure	Comparability	Assessment of outcome	Statistical test	Overall score (max=10)
1	Ahmed, S. M., et al. (2009)	*	*	*	**	-	**	*	8
2	Akkayagorn, L., et al. (2009)	-	-	-	*	-	**	*	4
3	Amini, M., et al. (2009)	*	-	-	*	-	*	*	4
4	Amnatsatsue, K. (2002)	-	-	-	**	-	**	*	5
5	Angkurawaranon, C., et al. (2015)	-	-	-	*	**	*	*	5
6	A-piwong Chalong (2011)	-	*	-	**	-	*	*	5
7	Aree-Ue Suparb & Monrudee Petlamul (2013)	*	-	-	*	-	*	*	4
8	Ar-Yuwat, S., et al. (2013)	*	*	-	**	-	*	*	6
9	Asawachaisuwikrom Wannipa (2003)	*	*	-	**	-	*	*	6
10	Asawachaisuwikrom, W. (2004)	*	*	-	**	-	*	*	6
11	Assantachai, P. and N. Maranetra (2003)	*	*	-	**	-	*	*	6
12	Assantachai, P., et al. (2006)	*	-	-	*	-	**	*	5
13	Atchara, P., et al. (2014)	-	-	*	**	-	*	*	5
14	Aungsusuknarumol Chaiya (2000)	*	*	-	**	-	*	*	6
15	Baiya, N., et al. (2014)	-	*	*	*	-	*	*	5
16	Banks, E., et al. (2011)	*	*	*	**	**	*	*	9
17	Banwell, C., et al. (2009)	*	*	*	*	-	*	*	6
18	Binhosen, V., et al. (2003)	*	*	*	**	*	*	*	8
19	Boonrin et al (2015)	*	*	-	*	-	*	*	5
20	Charoensook Kiattisak (2007)	*	*	-	**	-	*	*	6
21	Charoenying et al (2006)	*	-	-	**	-	*	*	5
22	Chawla N. and Panza A. (2012)	*	-	-	**	-	**	*	6
23	Chinuntuya Prapaporn (2001)	*	*	-	**	-	*	*	6
24	Chompaisal S. (1994)	*	-	-	*	**	*	*	6
25	Chongwatpol, P. and G. E. Gates (2016)	-	-	-	**	-	*	*	4
26	Chotikacharoensuk, P. (2002)	*	*	-	**	-	*	*	6

No.	Item	Sample representativeness	Sample size	Non-respondents	Ascertainment of exposure	Comparability	Assessment of outcome	Statistical test	Overall score (max=10)
27	Chuamoor, K., et al (2012)	-	-	*	*	-	*	*	4
28	Churangsarit, S. and V. Chongsuvivatwong (2011)	*	*	*	**	-	*	*	7
29	Dajpratham, P. and N. Chadchavalpanichaya (2007)	*	*	*	*	-	*	*	6
30	Dancy, C., et al. (2008)	-	-	-	**	**	**	*	7
31	Daraha, K. (2013)	*	-	-	*	-	*	*	4
32	Dasa Parinya (2001)	*	*	*	**	-	*	*	7
33	Decharat, S., et al. (2016)	-	-	-	*	-	*	*	3
34	Deenan et al (2001)	*	-	*	**	-	**	*	7
35	Ekpanyaskul, C., et al (2013)	*	*	*	*	-	*	*	6
36	Ethisan et al (2016)	-	-	-	**	-	*	*	4
37	Gidlof L. and Belay H.R. (2010)	*	-	-	-	-	*	*	3
38	Henry, C. J., et al. (2001)	-	-	-	**	-	*	*	4
39	Howteerakul, N., et al. (2006)	*	*	-	*	**	**	*	8
40	Ing-Arahm, R., et al. (2010)	-	-	*	**	-	*	*	5
41	Insawang, T., et al. (2012)	-	-	*	**	**	**	*	8
42	Intorn, S. (2003)	*	*	-	**	-	*	*	6
43	Ishimaru, T. and S. Arphorn (2016)	-	-	*	*	**	**	*	7
44	Jarupanich, T. (2007)	-	-	-	*	-	**	*	4
45	Jaruratanasirikul, S., et al. (2009)	*	-	*	**	-	*	*	6
46	Jermsuravong et al (2008)	*	*	*	**	-	*	*	7
47	Jirapinyo, P., et al. (1997)	-	-	-	**	-	*	-	3
48	Jitnarin, N., et al. (2008)	*	-	-	*	*	*	*	5
49	Jitramontree, N. (2003)	-	*	*	**	-	*	*	6
50	Julvanichpong Tanida (2015)	*	-	-	**	-	*	*	5
51	Junlapeeya, P. (2005)	-	*	*	**	-	*	*	6
52	Kabkaew Tatsanan (2006)	-	-	-	**	-	*	*	4
53	Kaewthummanukul et al (2008)	-	*	*	**	-	*	*	6
54	Kaewwit R. (2007)	*	*	-	**	-	*	*	6

No.	Item	Sample representativeness	Sample size	Non-respondents	Ascertainment of exposure	Comparability	Assessment of outcome	Statistical test	Overall score (max=10)
55	Kanchanomai, S., et al. (2012)	-	-	*	**	*	*	*	6
56	Kantachuvessiri, A., et al. (2005)	*	*	*	**	*	*	*	8
57	Keawvilai Somnuk (2009)	-	*	-	**	-	*	*	5
58	Khotcharrat, R., et al. (2015)	*	*	*	*	-	**	*	7
59	Khruakhorn, S., et al. (2010)	-	*	-	*	**	*	*	6
60	Khwanchuea, R., et al (2012)	-	-	-	*	**	**	*	6
61	Kiatrungrit, K. and S. Hongsanguansri (2014)	*	-	*	*	-	*	*	5
62	Kitrungpipat N. and Phannithit A. (2012)	-	*	-	*	-	*	*	4
63	Kittipimpanon (2006)	-	*	-	**	-	**	*	6
64	Klainin-Yobas, P., et al. (2015)	-	*	-	**	-	**	*	6
65	Kongcheewasakul et al (2014)	*	-	-	*	-	*	-	3
66	Konharn, K., et al. (2014)	*	-	-	**	-	**	*	6
67	Konharn, K., et al. (2015)	*	-	-	**	-	**	*	6
68	Kraithaworn, P., et al. (2011)	*	*	*	**	-	*	*	7
69	Kruavit, A., et al. (2012)	-	-	-	**	-	**	*	5
70	L, L. Y. L., et al. (2009)	*	*	*	*	-	*	*	6
71	Laosupap, K., et al. (2008)	*	*	-	*	-	*	*	5
72	Lavichant A. (2006)	*	*	-	**	-	*	*	6
73	Le et al (2006)	-	-	-	*	**	**	*	6
74	Leethong-in Mayuree (2009)	*	*	*	**	-	*	*	7
75	Limpawattana, P., et al. (2016)	*	*	-	**	**	**	*	9
76	Lindholm A. and Baylis R. (2009)	-	-	*	*	-	*	*	4
77	Mahanonda, N., et al. (2000)	-	-	-	*	-	**	*	4
78	Mongkhonsiri Pitsini (2007)	-	-	-	**	-	*	*	5
79	Morinaka, T., et al. (2012)	-	-	-	**	-	**	*	5
80	Mosuwan, L. and A. F. Geater (1996)	*	-	-	*	-	**	*	5
81	Mo-suwan, L., et al. (2014)	*	*	*	*	-	*	*	6
82	Nakhern P. and Kananub P. (2000)	-	-	*	**	-	*	*	5

No.	Item	Sample representativeness	Sample size	Non-respondents	Ascertainment of exposure	Comparability	Assessment of outcome	Statistical test	Overall score (max=10)
83	Nanakorn, S., et al. (1999)	-	-	-	*	-	*	*	3
84	Napradit, P., et al. (2007)	-	-	-	*	-	**	*	4
85	Narin et al (2008)	*	*	*	*	-	*	*	6
86	Ng, N., et al. (2009)	*	*	*	**	-	*	*	7
87	Ngamjaroen Annika (2005)	*	*	-	**	-	*	*	6
88	Nintachan, P. (2007)	*	*	*	**	-	*	*	7
89	Osaka, R., et al. (1999)	-	-	-	*	-	*	*	3
90	Othaganont, P., et al. (2002)	-	-	-	**	-	*	*	4
91	Page, R. M. and J. Suwanteerangkul (2009)	-	-	-	**	-	*	*	4
92	Page, R. M., et al. (2005)	-	-	-	**	**	*	*	6
93	Pancharean S. and Wanjan P. (2007)	*	*	-	**	-	*	*	6
94	Pasiri P. and Kuhirunyaratn P. (2015)	*	*	-	**	-	*	*	6
95	Pawloski, L. R., et al. (2010)	-	-	*	**	-	**	*	6
96	Peltzer, K. and S. Pengpid (2016)	*	-	-	**	-	*	*	5
97	Peltzer, K., et al. (2014)	*	-	*	**	**	*	*	8
98	Peltzer, K., et al. (2015)	-	-	*	**	-	*	*	5
99	Pengpid, S. and K. Peltzer (2013)	*	*	*	*	-	*	*	6
100	Pengpid, S. and K. Peltzer (2013)	*	*	*	*	-	*	*	6
101	Pengpid, S. and K. Peltzer (2015)	*	-	*	**	-	**	*	7
102	Pengpid, S., et al. (2015)	-	-	*	**	-	*	*	5
103	Pensri, P., et al (2010)	-	-	*	**	-	*	*	5
104	Piaseu, N., et al. (2001)	-	-	-	*	-	**	*	4
105	Pipatkasira, K. (2008)	*	*	-	**	-	*	*	6
106	Podang, J., et al. (2013)	-	-	-	**	**	**	*	7
107	Polin, S. (1999)	*	*	-	**	-	*	*	6
108	Pongchaiyakul, C., et al. (2004)	-	*	*	*	**	**	*	8
109	Poolsawat W. (2007)	*	*	-	**	-	*	*	6
110	Poomsrikaew Ornwanya (2011)	-	*	-	**	-	*	*	5

No.	Item	Sample representativeness	Sample size	Non-respondents	Ascertainment of exposure	Comparability	Assessment of outcome	Statistical test	Overall score (max=10)
111	Pornsakulvanich V. (2007)	*	*	-	*	-	*	*	5
112	Pothiban, L. (1993)	*	*	*	**	-	**	*	8
113	Prapimporn Chattranukulchai, S., et al. (2015)	-	-	-	*	-	**	*	4
114	Prombumroong, J., et al. (2011)	-	-	*	**	-	*	*	5
115	Rapheeporn, K., et al. (2013)	-	-	-	*	*	**	*	5
116	Razzaque, A., et al. (2009)	*	-	-	**	-	**	*	6
117	Rerksuppaphol, L. and S. Rerksuppaphol (2011)	*	-	-	*	-	*	*	4
118	Ruangdaraganon, N., et al. (2002)	*	*	*	**	-	*	*	7
119	Ruangrat, Achara. (2001)	*	*	*	**	-	*	*	7
120	Rungruang et al (2006)	*	*	-	-	-	*	*	4
121	Saithong et al (2004)	*	*	-	**	-	*	*	6
122	Samnieng, P., et al. (2013)	*	-	-	*	**	**	*	7
123	Sangthong, R., et al. (2012)	*	*	*	**	**	*	*	9
124	Siangsai C. and Sukonthasab S. (2015)	*	*	-	**	-	*	*	6
125	Siramaneerat I. and Sawangdee Y. (2015)	*	*	*	*	**	*	*	8
126	Sirikulchayanonta, C., et al. (2011)	*	*	-	**	**	*	*	8
127	Siriphakhamongkhon et al (2016)	*	*	*	*	-	**	*	7
128	Siripul, P. (2000)	*	*	*	**	**	**	*	10
129	Srichaisawat, P. (2006)	-	*	-	*	-	*	*	4
130	Sritara, C., et al. (2015)	-	-	-	**	-	**	*	5
131	Stewart, O. et al. (2014)	-	*	-	**	**	*	*	7
132	Sukrasorn, S. (2008)	*	*	-	**	-	*	*	6
133	Sumkaew, J. (2002)	-	*	*	**	-	*	*	6
134	Sumpowthong Kaysorn (2002)	*	-	-	*	-	*	*	4
135	Sutthajunya, C. (2003)	-	*	-	**	-	*	*	5
136	Suwanachaiy Sitamanats (2007)	-	*	-	**	-	**	*	6
137	Teparatana, C. (1997)	*	*	-	**	-	*	*	6
138	Thanakwang, K. (2009)	*	*	*	*	-	*	*	6

No.	Item	Sample representativeness	Sample size	Non-respondents	Ascertainment of exposure	Comparability	Assessment of outcome	Statistical test	Overall score (max=10)
139	Thasanasuwan, W., et al. (2016)	*	*	-	**	**	**	*	9
140	Thavillarp P. (2004)	*	*	-	**	-	*	*	6
141	Thongbai, W., et al. (2011)	*	*	-	**	*	*	*	7
142	Triprakong et al (2012)	*	*	-	**	-	*	*	6
143	Usman Y. (2004)	-	*	-	**	-	**	*	6
144	Vannarit Taweeluk (1999)	*	*	-	**	-	*	*	6
145	Vathesatogkit, P., et al. (2012)	*	-	-	**	**	**	*	8
146	Voraroon et al (2011)	-	*	*	**	-	*	*	6
147	Wakabayashi, M., et al. (2015)	*	*	*	*	**	*	*	8
148	Wannasuntad, S. (2007)	*	*	*	**	-	**	*	8
149	Watcharathanakij et al (2012)	*	*	*	**	-	*	*	7
150	Wattanapisit et al (2015)	-	*	*	**	-	*	*	6
151	Wattanapisit et al (2016)	-	-	*	**	-	*	*	5
152	Wattanasirichaigoon, S., et al. (2004)	*	-	*	-	-	*	*	4
153	Wattanasit Pissamai (2009)	*	*	*	**	-	*	*	7
154	Wichaidit, W., et al (2014)	*	*	*	**	**	**	*	10
155	Yamchanchai, W. (1995)	-	-	-	**	-	*	*	4
156	Yiammit Chanchalak (2013)	*	-	-	**	-	*	*	5
157	Youngpradith, A., et al. (2005)	*	-	-	**	-	*	*	5
158	เงินทอง ว. (Yuenthong et al) (2014)	-	-	-	*	-	*	*	3
159	เชยชม ก. (Cheychom Kongkiat) (2015)	*	*	-	**	-	*	*	6
160	ชลานุภาพ บ. (Chalanuphab Busarin) (2009)	-	*	-	**	-	*	*	5
161	ทองสุขนอก จ. (Thongsuknok et al) (2008)	-	*	-	**	-	*	*	5
162	นาคะ ข. N(Naka et al) (2002)	*	*	-	**	-	*	*	6
163	บุญรอง ผ. (Bunrong Pakamat) (2007)	*	*	-	**	-	*	*	6
164	พลนิล ศ. (Polnil Siwa) (2010)	-	*	-	*	-	*	*	4
165	พลรัตน์ น. (Polarat et al) (2004)	-	-	-	*	-	*	*	3
166	มากเจริญ ก. (Makcharoen Korarat) (2015)	*	-	-	**	-	*	*	5

No.	Item	Sample representativeness	Sample size	Non-respondents	Ascertainment of exposure	Comparability	Assessment of outcome	Statistical test	Overall score (max=10)
167	สุรกิจ จ, ธีรเวชเจริญชัย ส. (Surakij J. and Theerawejcharoenchai S.) (2007)	-	-	-	**	-	*	*	4
